# Distinct Cellular Origins and Differentiation Process Account for Distinct Oncogenic and Clinical Behaviors of Leiomyosarcomas

**DOI:** 10.3390/cancers15020534

**Published:** 2023-01-15

**Authors:** Elodie Darbo, Gaëlle Pérot, Lucie Darmusey, Sophie Le Guellec, Laura Leroy, Laëtitia Gaston, Nelly Desplat, Noémie Thébault, Candice Merle, Philippe Rochaix, Thibaud Valentin, Gwenaël Ferron, Christine Chevreau, Binh Bui, Eberhard Stoeckle, Dominique Ranchere-Vince, Pierre Méeus, Philippe Terrier, Sophie Piperno-Neumann, Françoise Collin, Gonzague De Pinieux, Florence Duffaud, Jean-Michel Coindre, Jean-Yves Blay, Frédéric Chibon

**Affiliations:** 1 INSERM U1218 ACTION, Institut Bergonié, 33000 Bordeaux, France; 2 CNRS UMR5800, LaBRI, 33400 Talence, France; 3 Department of Medical and Biological Sciences, Université de Bordeaux, 33000 Bordeaux, France; 4 OncoSarc, INSERM U1037, Cancer Research Center in Toulouse (CRCT), 31000 Toulouse, France; 5 Centre Hospitalier Universitaire (CHU) de Toulouse, IUCT-Oncopole, 31000 Toulouse, France; 6 Department of Pathology, Institut Claudius Régaud, IUCT-Oncopole, 31000 Toulouse, France; 7 Department of Medical and Biological Sciences, University of Toulouse 3, 31000 Toulouse, France; 8 Department of Medical Genetics, CHU de Bordeaux, 33000 Bordeaux, France; 9 Department of Oncology, Institut Claudius Régaud, IUCT-Oncopole, 31000 Toulouse, France; 10 Department of Surgical Oncology, Institut Claudius Régaud, IUCT-Oncopole, 31000 Toulouse, France; 11 Department of Oncology, Institut Bergonié, 33000 Bordeaux, France; 12 Department of Surgery, Institut Bergonié, 33000 Bordeaux, France; 13 Department of Pathology, Centre Léon Bérard, 69000 Lyon, France; 14 Department of Surgery, Centre Léon Bérard, 69000 Lyon, France; 15 Department of Pathology, Institut Gustave Roussy, 94800 Villejuif, France; 16 Department of Medical Oncology, Institut Curie, 75005 Paris, France; 17 Department of Pathology, Centre Georges-François Leclerc, 21000 Dijon, France; 18 Department of Pathology, Hôpital Universitaire Trousseau, 37170 Tours, France; 19 Medical Oncology Unit, APHM Hôpital La Timone, Aix Marseille University, 13000 Marseille, France; 20 Department of Pathology, Institut Bergonié, 33000 Bordeaux, France; 21 Department of Medical Oncology, Centre Léon Bérard, 69000 Lyon, France; 22 INSERM U1052, CNRS 5286, Centre Léon Bérard, Université Claude Bernard Lyon 1, 69000 Lyon, France

**Keywords:** leiomyosarcoma, differentiation, oncogenesis, transcriptional regulation, bioinformatics

## Abstract

**Simple Summary:**

Leiomyosarcomas are aggressive diseases mainly treated by surgical resection with or without conventional chemotherapy. Despite efforts to stratify patients, no targeted therapy nor immunotherapy has shown a major therapeutic effect. The oncogenesis of leiomyosarcoma is poorly understood, and its understanding would allow the detection of their weaknesses. By integrating large-scale data, we identified two specifically deregulated pathways involved in differentiation/proliferation switch (MYOCD/SRF and E2F1/RB1) in a subgroup of well-differentiated vascular smooth muscle cell-derived cells leiomyosarcomas. Targeting MYOCD/SRF interaction with a specific inhibitor decreased the viability of a cell line derived from this tumor subgroup, which makes this pathway a potential therapeutic target.

**Abstract:**

In leiomyosarcoma (LMS), a very aggressive disease, a relatively transcriptionally uniform subgroup of well-differentiated tumors has been described and is associated with poor survival. The question raised how differentiation and tumor progression, two apparently antagonist processes, coexist and allow tumor malignancy. We first identified the most transcriptionally homogeneous LMS subgroup in three independent cohorts, which we named ‘hLMS’. The integration of multi-omics data and functional analysis suggests that hLMS originate from vascular smooth muscle cells and show that hLMS transcriptional program reflects both modulations of smooth muscle contraction activity controlled by MYOCD/SRF regulatory network and activation of the cell cycle activity controlled by E2F/RB1 pathway. We propose that the phenotypic plasticity of vascular smooth muscle cells coupled with MYOCD/SRF pathway amplification, essential for hLMS survival, concomitant with PTEN absence and *RB1* alteration, could explain how hLMS balance this uncommon interplay between differentiation and aggressiveness.

## 1. Introduction

Cell differentiation is often associated with malignancy in solid tumors: in general, the more differentiated a tumor is, the less aggressive it is. Leiomyosarcoma (LMS), a rare (11% of adult soft tissue sarcomas [1]) and very aggressive (50% of patients relapse [2] with a median survival of 12 months) mesenchymal malignancy, challenges this concept. 

Previous studies [3,4,5,6,7,8,9,10] characterized a subgroup of well-differentiated LMS showing either equal [3,5,7], better [6,8,9], or worse [10] prognosis than the remaining LMS. These results relied on very different sample repositories (size, uterine LMS, metastatic and primary tissues), implying discrepancies between the reported subtypes. However, they demonstrated the existence of molecular subtypes that should be detected when considering treatment options. Current first-line treatment involves wide surgical resection for localized LMS or anthracycline-based chemotherapies for metastatic tumors since neither targeted therapy [11] nor immunotherapy [12] has demonstrated any significant therapeutic effects until now. However, developing targeted therapies in LMS is challenging since their oncogenesis is still poorly understood. However, genomic alterations are now well described, particularly the highly recurrent alterations of p53, RB1, and PTEN [10,13,14].

In the present study, we focus on these well-differentiated LMS and aim to understand how highly specialized cells keep or acquire their propensity to proliferate and migrate. In fine, we hope to detect potential therapeutic vulnerabilities, which could pave the way for a new treatment option. Moreover, previous studies highlighted the relatively homogeneous transcriptional behavior of well-differentiated LMS, which we suspect arises from a unique driver event, as reported by Watson and colleagues for other sarcomas with a strong chimeric driver oncogene [15]. We thus hypothesize that a common oncogenic event might underpin this homogeneous transcriptome. This could not only shed light on the biology of these LMS but also improve patient stratification and provide a therapeutic opportunity. 

## 2. Methods

### 2.1. Experimental Model and Subject Details

#### 2.1.1. Human Samples

Among samples used in the training cohort, 278 out of the 387 complex genetics sarcomas [16,17,18,19], the 60 GIST [20], and the 58 synovial sarcomas [21] are part of cohorts previously described (Table S1). All samples used in this cohort were part of the CRB-IB. In accordance with the French Public Health Code (articles L.1243-4 and R. 1243-61), the CRB-IB has received accreditation from the French authorities to use samples for scientific research. Samples used in the ICGC cohort were collected prospectively by the French Sarcoma Group as part of the ICGC program (International Cancer Genome Consortium). Clinicopathological data and patient information are summarized in Table S2. All cases were systematically reviewed by expert pathologists of the French Sarcoma Group according to the World Health Organization guidelines [22]. The Committee approved patients’ written informed consent for the Protection of Individuals. All samples were collected before treatment.

#### 2.1.2. Cell Lines and Primary Culture

All cell lines OC80 (LMS; Male), OC88 (LMS; Male), OC48 (LMS; Female), OC98 (UPS; Male), and OC110 (UPS; Female) were primary cultures established as previously described [23] and were cultured in RPMI-1640 (524000-025, Life Technologies, Carlsbad, CA, USA) supplied with 10% fetal bovine serum (S1810-500, Dutscher, Brumath, France). Cells were kept at 37 °C in a humidified chamber containing 5% CO_2_. 

### 2.2. Method Details

#### 2.2.1. Data Acquisition

##### Expression Microarray Data

According to the manufacturer's procedures, the 387 complex genetics sarcomas were analyzed on Human Genome U133 Plus 2.0 array (900466, Affymetrix, Santa Clara, CA, USA). For GIST, synovial sarcomas, LPS, and 87 complex genetics sarcomas, gene expression analysis was carried out by Agilent Whole Human 44K Genome Oligo Array (G4112A, Agilent Technologies, Santa Clara, CA, USA) according to the manufacturer’s protocol. 

##### Copy Number Data

CGH from the Affymetrix cohort (Table S1: Array-CGH in 53 cases was performed using the BAC-array as described in Chibon et al., 2010 [16], and with Genome-Wide Human SNP 6.0 arrays (901153, Affymetrix, Santa Clara, CA, USA) in 31 cases according to the manufacturer’s protocol with 500 ng DNA as input. 

##### Sequencing Data

DNA, total RNA, and miRNA were extracted from frozen samples of the ICGC cohort and sequenced using Illumina Technologies (Illumina Inc., San Diego, CA, USA) HiSeq2000 for DNA and RNA samples (paired-end) and HiSeq2500 for miRNA samples (single-end). Extraction, library preparation, and sequencing protocols are detailed in File S1.

#### 2.2.2. Sequencing Data Analysis

##### RNA Sequencing (RNA-Seq)

Alignment and expression quantification were performed as previously described in [19]. Fusion transcripts were detected with Defuse v0.6.1 [24] as previously described [25]. 

##### miRNA Sequencing (miRNA-Seq)

Reads were trimmed for adaptors using Cutadapt version 1.10 [26] with -q 30 and -m 18 parameters. Sequencing quality was assessed using FastQC version 0.11.8 from the Babraham Institute (https://www.bioinformatics.babraham.ac.uk/projects/fastqc/, accessed on 1 June 2019). We then aligned the reads with mature miRNA sequences from the miRbase Sequence database [27] according to the recommendations in [28]. We first used the BWA-aln algorithm version 0.7.17 [29] with -n 1 -o 0 -e 0 -k 1 -t 8 parameters and evaluated mapping quality using Qualimap version 2.2.2b [30]. We then used Samtools version 1.9 [31] to discard the reads with a mapping quality under 30 and to count mapped reads with the reference sequences (*samtools idxstats*).

##### Whole Genome Sequencing (WGS)

DNA reads were trimmed of the 5′ and 3′ low-quality bases (Phred cut-off 20, max trim size 30 nt), and sequencing adapters were removed with Sickle2 version 1.33 [32] and SeqPrep3 release 18 March 2016 (https://github.com/jstjohn/SeqPrep, accessed on 24 November 2022), respectively. Then, DNA-curated sequences were aligned using Bowtie v2.2.1.0 [33], with the very sensitive option, on the Human Genome version hg19. Thus, aligned reads were filtered out if their alignment score was less than 20 or if they were duplicated PCR reads, with SAMtools v0.1.19 [31] and PicardTools v1.118 [34], respectively. 

#### 2.2.3. Detection of Single Nucleotide and Structural Variants

##### Single Nucleotide Variant (SNV) 

SNV were detected in RNA-seq and WGS data using Samtools mpileup (SAMtools v0.1.19 [31]), with a minimum of 20 as Phred quality score (-Q 20), and bcftools (SAMtools v0.1.19 [35]) with options view –cvg for RNA-seq data and call –Am for WGS data. RNA-seq-detected variants with fewer than five coverage reads were filtered out. The variants detected in normal, tumor DNA, and tumor RNA were merged in the same file. Then, somatic variants were extracted with: (i) a minimum coverage of 14 reads in the tumor and 8 in the normal and (ii) a minimal allelic fraction of 0.3 in tumor and 0 in normal. Variants were annotated using the Annovar v20160314 tool [36]. Variants were selected whose alternative allele frequency (AF) in the Caucasian population (CEU) is lower than 0.1%, as reported in the 1000 Genome database [37]. Finally, variants were kept if localized in coding regions and were non-synonymous.

##### Structural Variants

Breakpoints were detected from WGS data. Paired-end reads were aligned using Bowtie v2.2.1.0 [33], a sensitive local option allowing soft-clipped sequences. The algorithm has three main steps: (i) identification of potential breakpoints, (ii) characterization of the second side of the breakpoints, and (iii) selection of high-confidence breakpoints. All parameters were set to analyze 60X tumor and 30X normal sequencing depth. Very conservative filters were used to minimize false positive detection. Details are available in File S1.

Copy number variants (CNV): Genome-Wide Human SNP 6.0 arrays were analyzed as previously described in [38]. Genes absent in more than a third of the patients were discarded. WGS-paired tumor/normal data from ICGC were processed using the cn.MOPS R package [39] with default parameters and a 500-nucleotide window. Regions were intersected with TxDb.Hsapiens.UCSC.hg19.knownGene R package version 3.2.2 [40] gene models. Regions with an estimated copy number of 128 were discarded.

For both datasets, genes overlapping segments with different predicted copy numbers were attributed with the lowest number of copies. 

#### 2.2.4. Experimental Validation

##### Fluorescent In-Situ Hybridization

FISH assay was performed on tissue microarrays using the Histology FISH accessory kit (K579911-2, Dako, Agilent Technologies, Santa Clara, CA, USA) according to manufacturer instructions. Thirty-eight tumors from the ICGC cohort were analyzed. Each case was represented by three spots 4 µm-thick and 1 mm in diameter. FISH assay was performed using a commercially available MYOCD FISH probe labeled in spectrum orange and a chromosome 17 control probe labeled in FITC (EG-MYOCD-CHR17-20ORGR, Empire Genomics, Williamsville, NY, USA). *MYOCD* and control probe enumeration was performed with a Nikon Eclipse 90i fluorescent microscope with appropriate filters. Pictures were captured using a Pannoramic 250 Flash II Digital Slide Scanner and analyzed with the Pannoramic Viewer (3DHISTECH Ltd., Budapest, Hungary). A case was considered interpretable when almost 80% of cells presented a signal for both probes. A loss was defined when only one copy of *MYOCD* was observed in the majority of cells; a normal status was when two copies of *MYOCD* were detected in the majority of cells; a gain or polysomy was when 3 to 5 copies of *MYOCD* or both *MYOCD* and the control probe were detected; and amplification was when the number of *MYOCD* signals was equal to or greater than 6, mainly when clustered signals were observed. 

##### Verification of Alterations 

For the ICGC cohort, *ATRX*, *TP53*, *RB1*, *PTEN*, and *DMD* sequences for each case obtained by whole genome sequencing were entirely screened using the Integrative Genomics Viewer (IGV version 2.6.3 [41]) to search for alterations possibly missed by the detection algorithms used. All SV were verified on gDNA by PCR and Sanger sequencing. MS/NS mutations were not found in either WGseq or RNAseq, and all FS were verified at both DNA and RNA levels by PCR and RT-PCR, respectively, followed by Sanger sequencing. For samples with enough material left, fusion transcripts detected by RNA-seq were verified by RT-PCR and Sanger sequencing. 

To screen mutations on genomic DNA, PCR primers were designed using the Primer 3 program version 0.4.0 [42] (https://bioinfo.ut.ee/primer3-0.4.0/, accessed on 1 June 2019). All PCR were performed on 50 ng of DNA using AmpliTaqGold^®^ DNA polymerase (4311820, Applied Biosystems, Foster City, CA, USA) according to the manufacturer’s instructions. PCR program for validating *TP53* mutations is described in [43]. For other PCR, the PCR program used was a Touch-down 60 °C program (TD 60 °C) (Hybridization temperatures: 2 cycles at a temperature of 60 °C, followed by two cycles at 59 °C, two cycles at 58 °C, three cycles at 57 °C, three cycles at 56 °C, four cycles at 55 °C, four cycles at 54 °C, five cycles at 53 °C and finally ten cycles at 52 °C).

Total RNA was first reverse-transcribed using random hexamers, and the High-Capacity cDNA Reverse Transcription Kit (4368814, Applied Biosystems, Foster City, CA, USA) according to the manufacturer’s instructions. All primers used were designed using the Primer 3 program version 0.4.0 [42] (https://bioinfo.ut.ee/primer3-0.4.0, accessed on 1 June 2019). All PCR was performed as previously described for PCR on genomics using the TD60 °C PCR program. 

##### Sanger Sequencing

PCR products were purified using an ExoSAP-IT PCR Purification Kit (US78200, GE Healthcare, Piscataway, NJ, USA). Sequencing reactions were performed with the Big Dye Terminator V1.1 Kit (4336805, Applied Biosystems, Foster City, CA, USA) according to the manufacturer’s recommendations. Samples were purified using the Big Dye XTerminator Purification kit (4376486, Applied Biosystems, Foster City, CA, USA) according to the manufacturer’s instructions, and sequencing was performed on a 3730xl Genetic Analyzer for cohort 1 or 3130xl Genetic Analyzer for cohort 2 (Applied Biosystems, Foster City, CA, USA). Sequences were then analyzed using the Sequencing analysis V5.3.1 and SeqScape V2.6 software (Life Technologies, Carlsbad, CA, USA). FinchTV software (V1.4.0) was also used (Geospiza, Seattle, WA, USA).

##### Immunohistochemistry

IHC assays were performed on tissue microarrays. IHC for PTEN detection was performed on a BenchMark Ultra instrument (Ventana, Washington, DC, USA). Antigen retrieval was performed using a CC1 protocol for 4 min at 100 °C (Ventana, Washington, DC, USA). The anti-PTEN antibody (1:200, 9559, RRID:AB_390810, Clone 138G6, Cell Signaling Technology, Danvers, MA, USA) was diluted in Prep kit 26 (783-2876, Roche, Basel, Switzerland) and incubated for 1 h. Antibody detection was performed with the Optiview detection kit for 12 min (860-099, Ventana, Washington, DC, USA). IHC for P53 detection was performed on a Bond-III (Leica Microsystems, Wetzlar, Germany) using the clone DO-7 monoclonal antibody (GA61661-2, Dako Omnis, ready-to-use, incubation 20 min, Agilent Technologies, Santa Clara, CA, USA). Antibody detection was performed using EnVision FLEX/HRP (GV80011-2, Dako, Agilent Technologies, Santa Clara, CA, USA). IHC pictures were taken with a Pannoramic 250 Flash II Digital Slide Scanner and analyzed with the Pannoramic Viewer (3DHISTECH Ltd., Budapest, Hungary). 

##### Immunofluorescence

Immunofluorescence was performed on tissue microarrays. First, tissues were de-paraffinized in three xylene baths for 5 min and rehydrated in successive baths of ethanol from 100% to 70%. For heat-induced epitope retrieval, slides were incubated for 20 min in a microwave oven in DAKO Target Retrieval pH6 (S203130-2, DAKO, Agilent Technologies, Santa Clara, CA, USA). Then, they were incubated with primary antibodies overnight at 4 °C in a humidity chamber and after with secondary antibodies for 1 h at room temperature. Slides were then mounted with Vectashield mounting medium plus DAPI (H-1200-10, Vector Laboratories, Burlingame, CA, USA). Images were acquired on a Zeiss Cell Observer Microscope (Zeiss, Oberkochen, Germany) or a confocal microscope LSM 780 (Zeiss, Oberkochen, Germany). The primary antibody used for Dp427 was the anti-human Dystrophin form Leica Biosystems (1:25, Dy4/6D3, NCL-DYS1, RRID:AB_442080, Leica Biosystems, Wetzlar, Germany) and the Dystrophin antibody from Abcam (1:100, ab15277, RRID:AB_301813, Abcam, Cambridge, UK) was used for all the dystrophin isoforms. Secondary antibodies used were goat anti-mouse Alexa Fluor 488 (1:400, A-11001, RRID:AB_2534069, Thermo Fisher Scientific, Waltham, MA, USA) and goat anti-rabbit Alexa Fluor 594 (1:400, A-11072, RRID:AB_2534116, Thermo Fisher Scientific, Waltham, MA, USA).

##### Cytotoxicity Analysis

LMS and UPS cells were seeded in 96-well microplates at a density of 2.10^3^ cells per well in 100 µL RPMI-1640 medium (524000-025, Life Technologies, Carlsbad, CA, USA). After 24 h incubation at 37 °C, 100 µL of medium was added with 2× of final concentration (100 to 1.5 µg/mL) of either CCG-1423 (10010350, CAS:285986-88-1, Bertin Bioreagent, Montigny le Bretonneux, France) or CCG-100602 (10787, CAS:1207113-88-9, Bertin Bioreagent, Montigny le Bretonneux, France). Cells were incubated at 37 °C for 72 h, after which 20 µL (5 mg/mL) solution of MTT (M2128, 5 mg/mL, Sigma, St. Louis, MO, USA) dissolved in water was added. After 2 h of incubation, media were removed, and the MTT metabolic product formazan was dissolved in 100 µL DMSO (5879, Sigma, St. Louis, MO, USA). Absorbance at 570 nm and 650 nm was measured with the Clariostar plate reader (BMG Labtech, Ortenberg, Germany), and cell viability was analyzed as follows: $((A_{sample\left(570nm\right)}-A_{sample\left(650nm\right)})/(A_{Control\left(570nm\right)}-A_{Control\left(650nm\right)}))*100$. IC_50_ was calculated with GraphPad Prism (GraphPad Software, San Diego, CA, USA) using non-linear regression. Each experiment was done three times in triplicate. 

#### 2.2.5. Quantification and Statistical Analysis

##### Normalization of Affymetrix and Agilent Micro-Arrays and Gene Selection

We used 87 samples analyzed on Agilent and Affymetrix platforms (Table S1). We selected the genes with Pearson’s correlation coefficient (PCC) with itself between chips over 0.8 or better than with any other genes in both experiments. We then normalized gene expression in separate experiments and then on a merged dataset by applying quartile normalization (preprocessCore R package version 1.48.0 [44]). We harmonized the expression between the platforms by gene expression median centering in each experiment and then added the mean of the experiment medians. The method is illustrated in Figure S1 and detailed in File S1.

##### Gene Module Clustering 

To define groups of co-expressed genes, we computed the pairwise Pearson’s correlation coefficient (PCC) of gene expression with variance > 2 across patients. We built a graph of co-expression with correlated genes (PCC > 0.7) and searched for communities using edge.betweenness.community from igraph R package version 1.2.5 [45]. 

##### Sample Clustering and PCA Analysis

In all cases concerning unsupervised clustering, samples were clustered using the *PCA* and *HCPC* functions from the FactoMiner R package version 2.3 [46]. They were visualized using the pheatmap R package version 1.0.12 [47]. PCA analysis on ICGC and TCGA miRNA transcriptome was computed using the *prcomp* R function and visualized using the ggbiplot R package [48]. We used the R package Rtsne version 0.15 [49] to visualize GTEX data with the parameters dims = 2 perplexity = 100 and max_iter = 1000.

##### Patient Classification

We computed centroids with citccmst R package version 1.0.2 [50] for hLMS and oLMS using 1672 differentially expressed genes detected in the Affymetrix cohort. Distance to centroids was computed as 1—Spearman’s correlation coefficient for each patient from all three cohorts. Patient classification was performed using the mclust R package version 5.4.6 [51]. We selected the Gaussian mixture distribution estimation that best fitted the hLMS centroid distance distribution (maximization of Bayesian Information Criterion).

##### Clinical Enrichment

Clinical enrichment significance was established using the two-tailed Fisher’s exact test for categorical data comparing one category to the others and Wilcoxon’s test for continuous data. 

##### Survival Analysis

Survival analysis was performed using the survival R package version 3.1-12 [52] by fitting a simple Kaplan-Meier model in which significance was set at log-rank test *p*-value < 0.01. Survival curves were plotted with survminer version 0.4.6 [53]. 

##### Differential Expression Analysis

mRNA: Differential expression (DE) analysis was performed using the two-tailed Welch Student’s test corrected for multi-testing using Holm’s method, and t-scores were stored as a measure of hLMS/oLMS expression. Expression of the *MYOCD* gene was considered high if its expression was over the third quartile of the global expression distribution (from all genes in all samples) separately in each experiment. 

ICGC miRNA: We applied the edgeR R package version 3.28.1 [54] on raw counts to normalize data and define significantly differentially expressed genes with a generalized linear model fitting (*glmQLFit*), correcting the returned *p*-values using Holm’s method. We kept as expressed any miRNAs (*filterByExpr*) with a summed raw count over all samples > 10 and represented by a minimum of five reads in at least one sample. 

TCGA miRNA: We computed the differential expression on TCGA miRNAs using the miRComb R package with the *limma* method [55]. We kept miRNAs with a median normalized count >1 in at least one of the LMS groups in both cohorts, having an hLMS/oLMS absolute logFC > 1, and a Holm’s adjusted *limma p*-value < 0.01. We used the *lm* function from the stats R package to compute the R^2^ value between ICGC and TCGA logFC. 

##### Functional Enrichment and Mapping

Modules of co-expressed genes were analyzed using the *enricher* function from the ClusterProfiler R package version 3.14.3 [56] with the Molecular Signatures Database version v6 (MSigDB) [57]. Significance threshold of the hypergeometric test FDR adjusted *p*-value was set at <0.05.

Differentially expressed genes were analyzed using the command line version of the GSEA software (version 3.0) [57] and MSigDB v6. We submitted the gene list ranked by hLMS/oLMS t-scores to the *xtools.gsea.GseaPreranked* function with default parameters. The significance threshold on the permutation test FDR-adjusted *p*-value was set at 0.05. For clarity, only terms with adjusted *p*-values < 0.01 are reported. Enrichments for regulatory elements in groups of over- and under-expressed genes were performed on the iCistarget webserver (https://gbiomed.kuleuven.be/apps/lcb/i-cisTarget/, accessed on 2 July 2020) [58]. The significance threshold of the normalized enrichment score (NES) was set at >3 by default. Annotations related to position weight matrix predictions from the same transcription factors were grouped.

We used the GSAn webserver (https://gsan.labri.fr/, accessed on 24 August 2020) [59] with default parameters to exhaustively annotate genes with the most precise Gene Ontology term. 

##### miRNA-mRNA Interaction Analysis

The MiRComb R package [55] was used to integrate miRNA, mRNA expression data, and experimentally validate miRNA-mRNA interactions from miRecords v4 [60] and miRTarBase v7.0 [61]. We retrieved 6262 known interactions representing interactions between 30 DE pre-miRNAs and 3850 genes. Pre-miRNA expression was estimated by averaging signals from derived mature miRNA. We kept interactions for which mRNA and miRNA had an hLMS/oLMS absolute log Fold Change (logFC) > 1, a *limma p*-value < 0.01, a significant Pearson’s anti-correlation (adjusted *p*-value < 0.01), and were described in at least one of the two databases. 

##### CNV Recurrence Analysis

Alteration recurrence was estimated by computing the frequency of each event (homozygous, heterozygous deletion, gain of one copy and amplification, i.e., gain of four copies or more), i.e., the number of a given event divided by the total number of patients (missing data being discarded). To evaluate alteration enrichment in each LMS group, losses were grouped into homo- and heterozygous deletions and gains and amplifications (one-tailed Fisher’s exact test *p*-value < 0.01). We computed enrichment for each type of event in the 291 cytobands by comparing the number of significantly altered genes defined above for each band (one-tailed Fisher’s exact test corrected with Holm’s method < 0.01). If more than one type of event was enriched, the most significant was kept.

##### Mutational Pattern Analysis

We analyzed patterns of somatic mutation using the MutationalPatterns R package version 1.12.0 [62]. We first generated a 96 tri-nucleotide mutation count matrix per patient which we compared to the 30 COSMIC signatures v3.1 [63]. We kept signatures with cosine similarity > 0.75 and computed the optimal contribution that best explained the observed mutational profiles in patients.

Tumor mutation burden was computed using the total number of somatic variants divided by the total length of human genome version hg19 (22 autosomal and 2 sexual chromosomes). 

## 3. Results

### 3.1. Identification of a Group of 42 LMS Behaving as Simple Genetic Sarcomas 

To detect LMS molecular subtypes within sarcoma samples, we combined micro-array datasets obtained on Affymetrix (387 complex genetic sarcomas including 98 LMS) and Agilent platforms (60 GIST, 58 synovial sarcomas, 50 LPS, and 87 complex genetic sarcomas) (Figure S1A, total = 555 samples). We selected 9066 genes (out of 17,854 genes common to both platforms, Figure S1B), showing enough consistency to merge and normalize all datasets (see methods and Figure S1C). 

**Figure 1:**
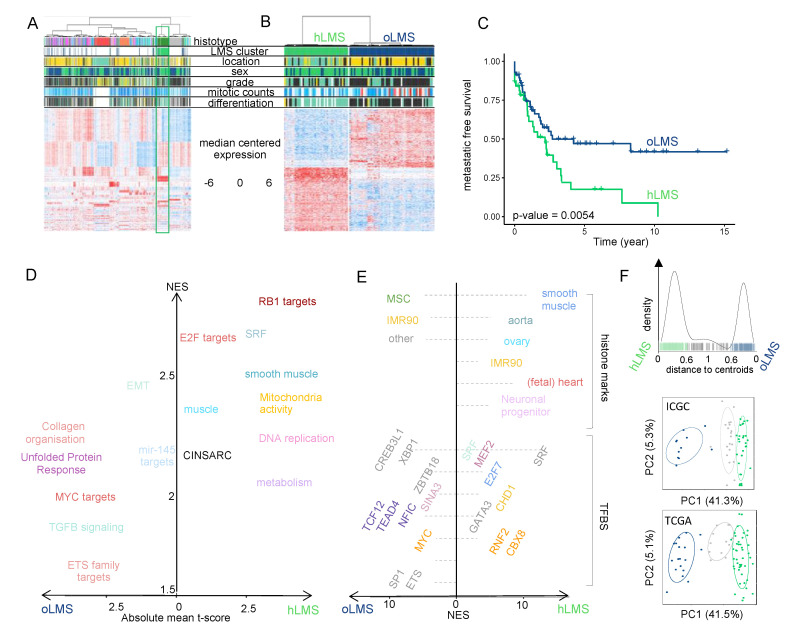
Transcriptional analysis and patient classification: (**A**,**B**). Heatmaps showing clustering of 555 sarcoma patients and 455 genes (**A**) and 98 LMS patients and 1672 differentially expressed genes between hLMS and oLMS (**B**). Patients were clustered using HCPC method, and genes were grouped by co-expression modules. Color schemes. Histotype: green forest: leiomyosarcomas, red: GIST, pink: undifferentiated sarcomas, orange: myxoid liposarcomas, blue: dedifferentiated sarcomas, grey: synovial sarcomas, turquoise: other sarcomas. Location: yellow: extremities, green: internal trunk, black: trunk wall. Sex: green: female, blue: male. Grade and differentiation: yellow: 1, green: 2, black: 3. Mitotic count: blue to red: from low to high: A—0 to 120, B—0 to 60. Cluster: green: hLMS, blue: oLMS. (**C**). Kaplan-Meier metastasis-free survival analysis in hLMS and oLMS. The number indicates the Log-Rank test *p*-value. (**D**). GSEA analysis on z-scores obtained from hLMS/oLMS gene expression comparison. Each dot is an enriched term (FDR < 0.01); size corresponds to the number of genes involved; the *x*-axis contains the mean t-score of all genes annotated in the given term, and the *y*-axis corresponds to the GSEA NES score. Related terms are colored the same way. (**E**). i-Cistarget analysis of 843 under- and 800 over-expressed genes in hLMS relative to oLMS. The *x*-axis represents the NES score obtained for over-expressed genes from 0 to the right and under-expressed genes from 0 to the left. The left and right parts are independent; the enriched features were clustered on the *y*-axis according to the cell type or tissue they were analyzed from. Histone modifications are only active transcription marks (H3K4me1, H3K4me3, H3K27ac, and H3K9ac). Detailed legends for E-F are available in Table S3C,D, respectively. (**F**). Top panel: distance distribution to centroids (*x*-axis) computed from transcriptional signature for ICGC and TCGA patients (bars on the *x*-axis). Colors correspond to cluster assignation: patients with a distance lower than 0.6 to one of the centroids were assigned to the corresponding centroid (green: hLMS, dark blue: oLMS), while patients with intermediate value were not classified (light grey). Middle and bottom panels: PCA analysis using transcriptional signature genes in ICGC and TCGA cohorts. Each point is a patient, green: hLMS, dark blue: oLMS, and light grey: not classified. *x*-axis and *y*-axis represent principal components 1 and 2 and their associated variance representation, respectively.

We assumed that selecting modules of co-expressed genes that potentially group genes with similar functions would lead to more meaningful patient clustering. We detected 15 co-expression modules (out of 54) carrying at least five genes from 455 highly correlated genes. Thirteen modules were significantly associated with biological functions and cellular components (e.g., immune system activation, cell cycle, skeletal muscle or smooth muscle-related, adipogenesis, extracellular matrix, apical plasma membrane, genomic positional bias, Table S3A, Figure S1D). We used these 54 modules in a non-supervised approach to cluster the 555 samples and observed a subgroup of LMS clustering while the other LMS were mixed with other pleomorphic sarcomas. This LMS subgroup appeared to behave like sarcomas with a recurrent alteration, i.e., with a relatively homogeneous transcriptomic program driven by a strong oncogene [15], as observed with GIST, myxoid liposarcomas and synovial sarcomas (Figure 1A). We thus hypothesized that this LMS subgroup (41 patients out of the 98 LMS) could be driven by a strong oncogenic program reflected by this specific gene expression profile. 

To select genes that best characterized these LMS, we compared them with the remaining 57 LMS mixed with the other sarcomas. As the 98 LMS were all analyzed on the Affymetrix chip, we used the 22,635 genes in the chip. We identified 1672 differentially expressed genes (Table S4A) that we used to re-cluster the samples. Almost all samples were classified similarly (95/98) regarding the analysis performed above on the 555 samples. We obtained 42 homogeneous LMS (hLMS) and 56 other LMS (oLMS) (Figure 1B). 

### 3.2. hLMS Are Intra-Abdominal, Low-Grade, Metastatic LMS with Homogeneous Transcriptional Behavior

After confirming that gene expression profiles within hLMS were significantly more homogeneous than within the other group (Wilcoxon’s test; *p* = 2.9 × 10^−13^), we tested clinical feature enrichments (Table 1). hLMS were primarily located in the abdominal cavity (*p* = 8.5 × 10^−9^), developed in females (*p* = 0.003), were well differentiated (*p* = 3.9 × 10^−9^) and consequently were more frequently grade 1 or 2 (low grades, *p* = 5.5 × 10^−4^). Interestingly, despite this differentiation and grading, they had a poorer prognosis than oLMS (*p* = 0.0054, Figure 1C).

### 3.3. The Transcriptional Signature Highlights Cell Cycle and Differentiation Pathways Specific to LMS Subgroups 

Functional enrichment analysis of differentially expressed (DE) hLMS/oLMS genes (Figure 1D and detailed in Table S3B,C) revealed biological differences between the two groups. The transcriptional program in hLMS is strongly associated with smooth muscle cell and cell cycle activity, as evidenced by the enrichment of *SRF*, *E2F,* and *RB1* targets, CINSARC signature, DNA replication, metabolism, and mitochondrial activity in up-regulated genes. In line with these results, activating marks (H3K4me1, H3K27Ac, and H3K9Ac) from ChiP-seq experiments in smooth muscles (stomach, rectum, colon, aorta) as well as ChiP-seq peaks for *SRF* and *MEF2A* were enriched in over-expressed hLMS genes (Table S3D, Figure 1E). On the other hand, oLMS were associated with unfolded protein response-related terms, epithelial-mesenchymal transition, and the TGFβ signaling pathway. At the same time, enriched histone marks in over-expressed oLMS genes were found to be comparable to those in fibroblasts, epithelial and derived mesenchymal stem cells. These genes are under the regulation of transcription factors (TF) like MYC, ETS1, or ELK1. Therefore, we hypothesize that hLMS and oLMS originate from distinct cell types. To investigate this hypothesis, we stratified patients from two independent multi-omic cohorts.

### 3.4. Gene Signature Identifies hLMS in Two Independent Cohorts

To classify LMS from the ICGC (59 patients) [64] and TCGA (75 patients) [13] cohorts, we computed the distance to hLMS and oLMS centroids based on the expression of the 1672 DE genes from the Affymetrix cohort. When the cohorts were merged, 102 cases were strongly correlated enough with one centroid (Figure 1F), classifying 73 as hLMS and 29 as oLMS. Computation of clinical enrichment showed hLMS to be mainly intra-abdominal (*p* = 1.5 × 10^−7^), well differentiated (*p* = 1.8 × 10^−5^), carried by women (*p* = 0.007), and with homogeneous transcriptional profiles (*p* < 2.2 × 10^−16^) (Table 1), consistent with information from the training cohort. However, we observed no difference in metastasis-free survival between the two groups.

### 3.5. hLMS Originate from Vascular Smooth Muscle Cells

**Figure 2:**
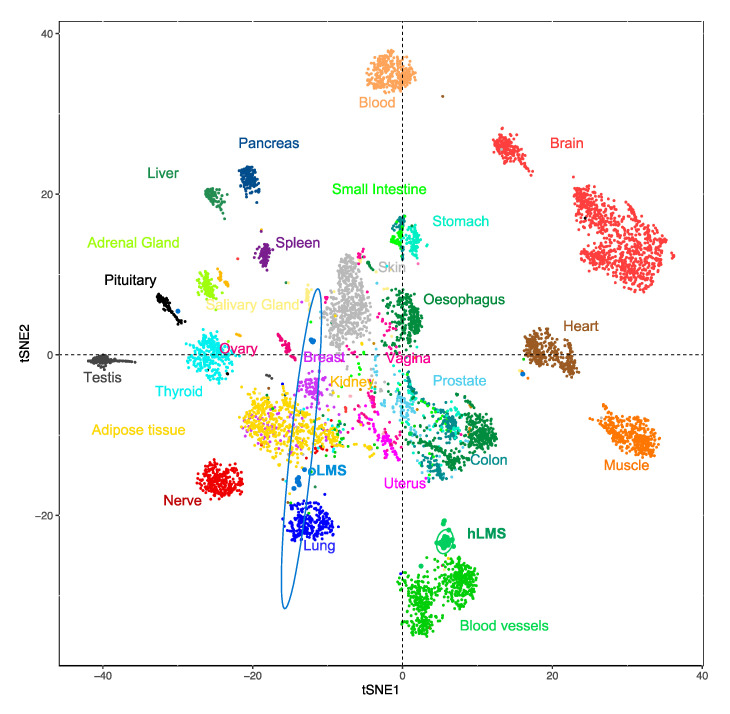
t-SNE clustering from most expressed hLMS 100 genes in 7414 normal and LMS samples from the GTEX dataset. Each point represents a sample, and the color code corresponds to the tissue type.

To investigate the potential origin of hLMS, we analyzed the 100 most expressed hLMS genes in 7414 samples from 30 different normal tissues (TCGA GTEX dataset). Using a t-SNE approach, we observed that these genes allowed normal samples to be grouped mainly according to their tissue of origin (Figure 2). Visceral smooth muscle tissues were mixed and separated from blood vessels to which the hLMS were the closest. hLMS and oLMS were well separated, and oLMS showed a more comprehensive distribution between lung, adipose, and breast tissues. These results support our hypothesis that the two LMS groups have a different origin and suggest that hLMS could originate from vascular smooth muscle cells (vSMC). 

We annotated these 100 genes (Figure S2) using GSAn [59] (Table S3E). We found that 50 of them are part of the extracellular exosome, which are molecules (mRNA or proteins) exported to the extracellular space. This highlights the role of the extracellular matrix (ECM) and cell-to-cell communication in hLMS pathology. Cell differentiation and migration were represented by 32 and 24 genes, respectively, which suggests the co-existence of both contractile (*MYH11*, *CNN1*, *MYL9*, *LMOD1*) and synthetic (*FN1*, *TNC*, *COL1A1/2*, *MSN*, *MFAP4*) phenotypes in hLMS.

We used multi-omics to analyze two additional LMS cohorts to complete our analysis of the genomic differences between hLMS and oLMS.

### 3.6. miRNAs Adopt Specific Behavior in hLMS

**Figure 3:**
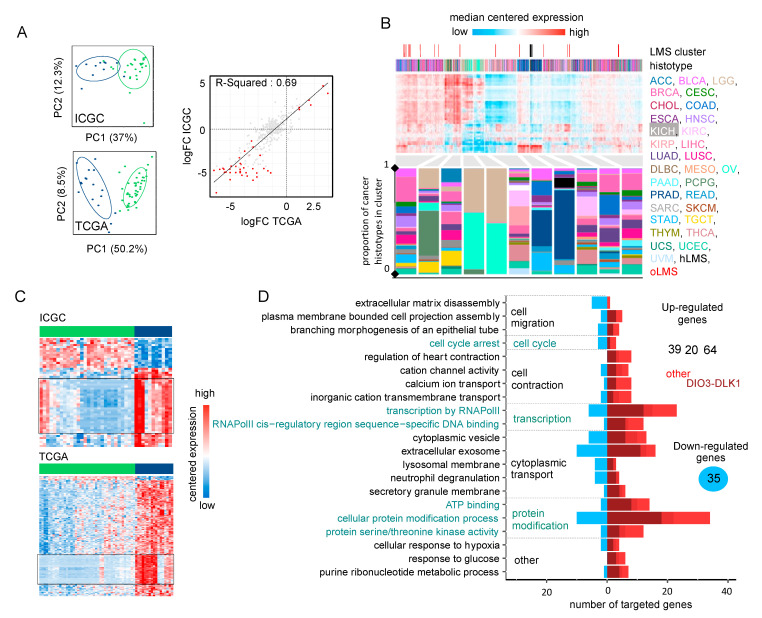
Analysis of miRNA expression (**A**). Left panel: PCA obtained from the expression of 484 mature miRNAs in 39 ICGC patients (top) and 475 mature miRNAs in 60 TCGA patients (bottom). Colors correspond to hLMS (green, ICGC: 28 patients, TCGA: 43 patients) and oLMS (blue, ICGC: 11 patients, TCGA: 17 patients). The first two principal components are shown with the percentage of variance they capture. Right panel: Scatterplot showing correlation between hLMS/oLMS Log Fold Change (LogFC) in ICGC (*y*-axis) with TCGA (*x*-axis). Each dot represents a mature miRNA (347 expressed in both cohorts), and red indicates 71 significant mature DE miRNAs in both cohorts. The line represents linear regression with interval confidence in shaded grey. (**B**). HCPC clustering on the 41 mature miRNAs differentially expressed in LMS subtypes across the 9564 PANCAN samples. Heatmap showing median-centered miRNA expression (low: blue to high: red). Column annotations represent the histotype of samples (bottom) and focus on LMS clusters (top) for which colors are specified at the bottom of the figure. Composition in the histotype of clusters is detailed in the bar plot below the heatmap. The *y*-axis corresponds to the proportion. (**C**). Heatmap showing differentially expressed miRNAs (rows) in ICGC (top, 55 miRNAs) and TCGA (bottom, 243 miRNAs). Column annotation corresponds to hLMS (green) and oLMS (blue). Expression values are median-centered (low: blue to high: red). Black rectangles highlight mature miRNAs from the DIO3-DLK1 miRNA cluster (ICGC: 26, TCGA: 63, 25 in common). (**D**). Functional terms mapped to 158 miRNA-targeted genes. The *x*-axis indicated a number of down-regulated (toward left in blue) and up-regulated (toward the right in dark red if targeted with only miRNAs from the DIO3-DLK1 cluster, medium red if targeted by both miRNAs from DIO3-DLK1 cluster and other miRNAs and light red if targeted by other miRNAs) genes annotated with the term (*y*-axis).

We analyzed 475 expressed mature miRNAs from the 39 patients in the ICGC cohort (28 hLMS and 11 oLMS) and 453 in the 60 TCGA patients (43 hLMS and 17 oLMS). PCA analysis performed with all expressed miRNAs strongly differentiated hLMS and oLMS along the first principal component, which explains 37% (ICGC) and 50.2% of the variance (TCGA) (Figure 3A). The high correlation (R^2^ = 0.69, Figure 3A) between hLMS/oLMS log-fold changes from both cohorts indicates that each group identified independently in each cohort is consistent and represents two groups of similar diseases. The results of DE analyses of both cohorts are presented in Table S4B. We used the TCGA pan cancer (PANCAN) dataset to test our hypothesis that the two groups have a different cellular origin. To this end, we used the 41 significantly differentially expressed miRNAs (35 under-expressed and six over-expressed in hLMS) to classify all the cancer samples (Figure 3B). All hLMS clustered together among 467 samples mainly from prostate adenocarcinomas (65%), digestive tract tumors (stomach, colon, esophagus, rectum: altogether 14%), LMS (13 gynecological, 8 unclassified: together with hLMS, 13.7%). 

Interestingly, the eight most discriminative miRNAs of the cluster containing hLMS were four over-expressed (*MIR143-3p*, *MIR145-3/5p*, and *MIR1*) and four under-expressed (*MIR455-3p*, *MIR503-5p*, and *MIR424-3/5p*) miRNAs in hLMS that are involved in vascular smooth muscle phenotypic modulation [65,66,67,68,69]. These results corroborated our hypothesis of a smooth muscle origin of hLMS, unlike oLMS, which was spread across several clusters. 

Intriguingly, all 87 mature miRNAs located in the DLK1-DIO3 imprinted genomic region on chromosome 14 (14q32) were repressed in hLMS. Indeed, 25 miRNAs are among the 35 significantly down-regulated in hLMS (highlighted in Figure 3C), 20 others show negative log-fold changes (Figure S3A) with very low expression in hLMS (Figure S3B), and 42 were not detected in any LMS groups. To evaluate the specificity of this global repression, we compared the expression profiles of the 72 miRNAs (among the 87 DLK1-DIO3) present in the PANCAN dataset. Most hLMS (37/42) clustered within a group of 563 patients, representing 6% of all samples, preferentially with kidney (37%), thyroid (23%), eye (11%) carcinomas, and sarcomas (5.5%, 13 gynecological LMS, one unclassified LMS, two oLMS, seven UPS, six myxofibrosarcomas, three dedifferentiated liposarcomas) (Figure S3C). hLMS thus cluster with cancer deriving from diverse cell types, suggesting an uncommon repression that might be due to a specific mode of oncogenesis rather than a vSMC origin.

To evaluate the putative impact of dysregulated miRNAs on hLMS biology, we analyzed their post-transcriptional regulatory network by integrating mRNA and miRNA expression data. We found 210 significant miRNA-mRNA interactions predicted in both ICGC and TCGA cohorts (negative Pearson’s correlation coefficient (PCC), adjusted *p* < 0.01) and present in at least one database (Table S4C). We annotated the 158 corresponding target genes (35 down- and 123 up-regulated in hLMS) with GSAn [59]. Twenty-one terms with high specificity were mapped, and none was specific to the DIO3-DLK1 miRNA cluster, up- or down-regulated target genes, except “response to glucose” represented only by up-regulated genes (Figure 3D, detailed in Table S3F). Dysregulated genes are implicated in significant pathways, such as cell migration (“plasma membrane-bound cell projection assembly”, “extracellular matrix disassembly”), cell contraction (“regulation of heart contraction”, “cation channel activity”, “calcium ion transport”), cell cycle and transcriptional regulation. 

hLMS miRNA profiles are highly homogeneous and appear closely related to vSMC phenotypic modulation, and the predicted regulatory network shows characteristics of both contractile, migratory, and proliferative phenotypes. On the other hand, oLMS are heterogenous with no specific program. The question, therefore, arose whether these two types of LMS also have a distinct genomic mode of oncogenesis.

### 3.7. hLMS Show Recurrent and Specific Genomic Instability

**Figure 4:**
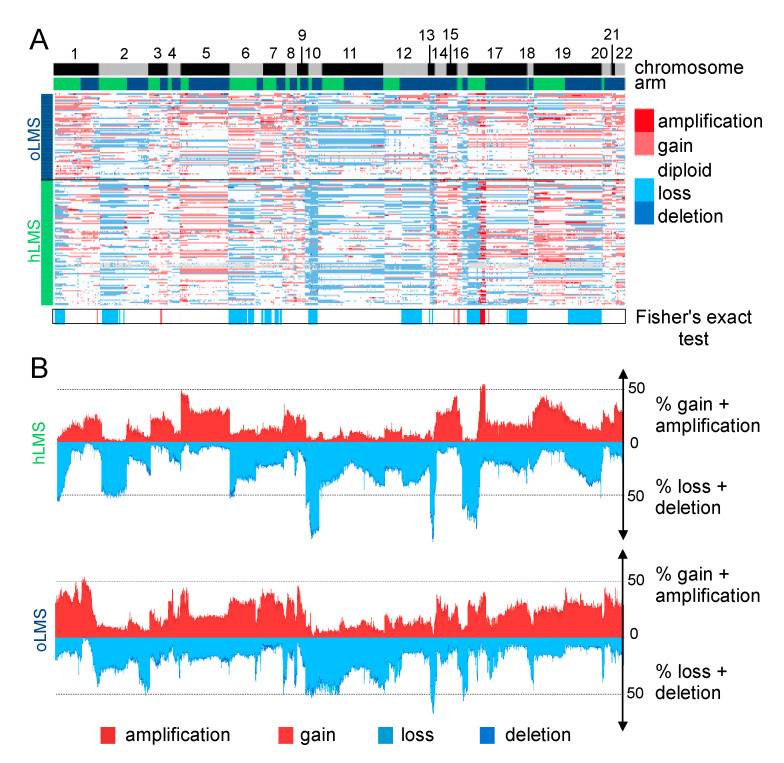
Copy number analysis of 7479 genes in LMS merged cohort (84 Affymetrix (CGH), 39 ICGC (WGS), and 62 TCGA (CGH) patients). (**A**). Heatmap showing copy number of genes (columns) in each patient (rows). Patients are grouped according to LMS type (hLMS: green, oLMS: blue). Annotations above the heatmap show chromosomes from 1 to 22 alternating grey and black and arms (p: green, q: blue). The annotation below shows significantly enriched events in hLMS, Fisher’s Exact test *p*-value < 0.01. The color scheme is the same for copy number and enrichment: homozygous deletion: dark blue, heterozygous deletion: light blue, normal: white, light red: gain of one copy, dark red: gain of 4 or more copies. (**B**). Penetrance plot. The percentage (*y*-axis) of gain (red) and loss (blue) events are represented in hLMS (top panel) and oLMS (bottom panel). Each position on the *x*-axis is a gene that corresponds to genes in A.

LMS are characterized by their highly rearranged genome [17]. Copy number alterations in hLMS appeared more homogeneous than in oLMS in the merged cohort (Figure 4A,B) and between the cohorts, indicating highly correlated penetrance profiles (Figure S4A). 

**Figure 5:**
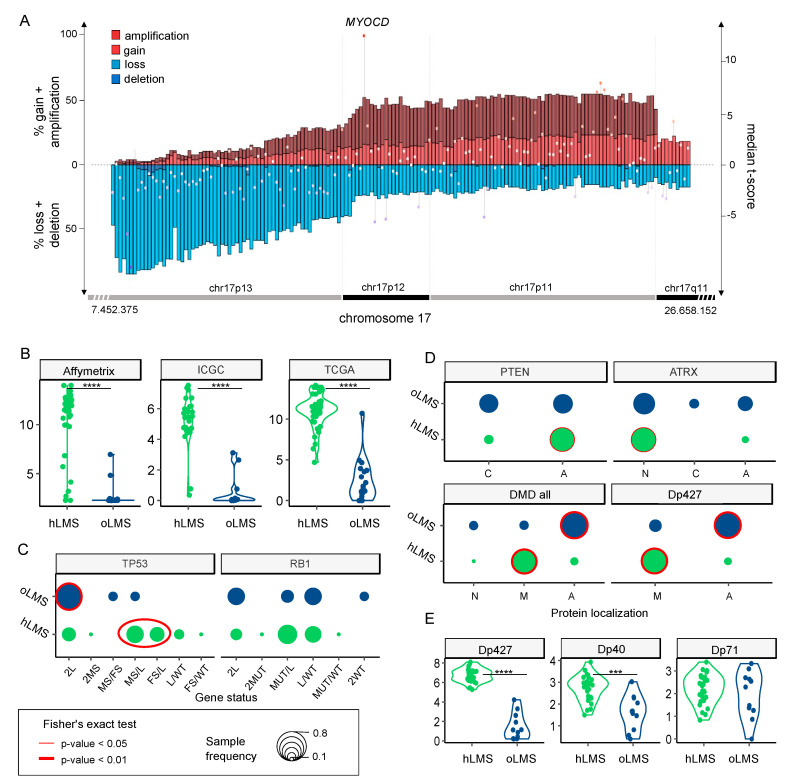
Zoom on genes of interest. (**A**). Zoom on chr17p13-11/q11 genomic region (*x*-axis) penetrance profile containing *MYOCD*. The left *y*-axis indicates the percentage of loss (light blue), deletion (dark blue), gain (light red), and amplification (dark red); the right *y*-axis shows hLMS/oLMS median t-scores (from the three cohorts). Each gene is represented by the bar (penetrance) and dot (t-score). (**B**,**E**). Violin plots showing expression of *MYOCD* gene and *DMD* isoforms (RNA level, *y*-axis) in hLMS and oLMS in the three cohorts, respectively. **** indicates a *t*-test *p*-value < 10^−7^, *** *p*-value < 10^−3^. (**C**). Distribution of *TP53* and *RB1* allele status in hLMS and oLMS. Dot sizes correlate with the percentage of patients in the LMS group harboring defined status. Cases with biallelic inactivation of *TP53* are compared between groups (2L + 2MS: only one mechanism altering both alleles versus MS/FS + MS/L + FS/L: two different mechanisms altering each allele): red oval indicates Fisher’s test *p*-value < 0.01. L: loss, MS: missense, FS: false sense, MUT: MS or FS, WT: wild-type; if 2 is specified, both alleles are concerned. (**D**). Cellular distribution of PTEN, ATRX, and DMD proteins in hLMS and oLMS. For DMD, localization of its Dp427 isoform is also presented. A: absent, N: nuclear, M: membranous, C: cytoplasmic. Dot sizes correlate with the percentage of patients in LMS group harboring defined localization. The red circle indicates Fisher’s Exact test *p*-value < 0.01 (bold) and <0.05 (thin).

Recurrent alterations were significantly enriched in hLMS, especially amplification of chromosome 17p12-p11.2 and loss of chr10q, chr13q14, and chr17p13 (Figure 4B and Table S5A). The chr17p12-p11.2 amplified region was not only significantly enriched in hLMS, with 31% of hLMS showing an amplification versus 7–8% of oLMS but was also the most frequently amplified region in hLMS (Figure 5A, Table S5A). Among the genes located in this region, *MYOCD* was the most frequently amplified (36% of hLMS) and was the most over-expressed gene in this region in hLMS compared to oLMS (*p* < 10^−7^ all cohorts considered, Figure 5A,B, Table S5B). *MYOCD* expression was very high in 84% of hLMS (97/115, detailed per cohort in Figure S4B), whereas it was not expressed or at a very low level in oLMS, even in those with a gain or an amplification (Figure 5B, Figure S4C and Table S6A). Interestingly, the well-known tumor suppressors *RB1*, *PTEN* and *TP53* belong to three of the eight most significantly enriched lost regions in hLMS: chr13q14 (88% versus 72%), chr10q23 (87% versus 66%) and chr17p13 (69% versus 13%) respectively (all cohorts considered) (Figure 4, Table S5A,B).

When we used whole genome characterization of the ICGC to investigate these genetic variations further, we found that oLMS tended to be more rearranged (*p* < 0.05, Figure S4D) than hLMS but that the mutational burden was similar between them (*p* = 0.5, Figure S4D). While no COSMIC mutational signature could be associated with the LMS groups, we found a patient-specific predicted contribution of signatures mainly related to defective DNA repair, except for LMS23, which had a disproportionate mutational burden (120 mutations/Mb versus less than one mutation/Mb for the other), and a mutational profile similar to ultraviolet light exposure, which is coherent with its location on the scalp. (Figure S4E). Very few genes were identified as recurrently mutated (SNV). However, by combining the different alterations, i.e., mutations, structural variants (SV), and losses, we found very frequently altered genes across all ICGC-LMS: *TP53* altered in 100% of cases, *RB1* in 97.4%, *PTEN* in 82%, *ATRX* in 28.2% and *DMD* in 25.6%. (Table 2 and Table S6).

We found no significant difference between the two LMS groups in RB1 and TP53 global alteration frequencies. However, *TP53* presented significantly different alteration patterns. oLMS preferentially lost *TP53* completely (9/11, 82%), whereas 64.3% of hLMS (18/28) exhibited various alterations on each allele with losses, missense, and frameshift mutations (Fisher’s exact test, *p* = 0.01, Figure 5C, Table S6A). The same trend was observed for *RB1* without reaching significance (Figure 5C, Table S6A). 

*PTEN* was almost exclusively altered by complete gene deletion, regardless of the LMS type (Table 2 and Table S6A). However, although 82% of cases in both groups were changed, its protein expression loss was significantly associated with hLMS (Figure 5D).

*ATRX* mutations are described in detail in [64], in which we reported their characterization in the whole ICGC cohort (including the 39 LMS studied here). We showed that *ATRX* alteration and ATRX protein expression loss are associated with uterine LMS and the oLMS type [64]. Accordingly, ATRX nuclear localization was significantly enriched in hLMS (Table S6A, Figure 5D).

*DMD* tended to be more frequently altered in oLMS than in hLMS (45.4% and 17.8%, respectively) (Table 2). Most *DMD* alterations involved SV, mainly affecting the long isoforms (Table S6A,C). Regardless of *DMD* genomic status, *Dp427m*, its muscle-specific transcript isoform, was significantly less expressed in oLMS (*p* = 1.2 × 10^−9^), as was *Dp40* (*p* = 1.9 × 10^−4^). On the other hand, the expression of *Dp71*, a ubiquitous isoform, was similar in both LMS types (*p* = 0.63) (Table S6A, Figure 5E). Results were confirmed at the protein level, with a significant association of global DMD expression loss, particularly of Dp427 in the oLMS type (Figure 5D). 

Therefore, despite having similar alteration frequencies of the two significant suppressor genes *TP53* and *RB1*, the mechanistic differences and specific expression enrichments of the two LMS types suggest that their oncogenic processes differ. The main features underpinning this distinction are the amplification and strong expression of *MYOCD* and the loss of PTEN protein in hLMS. Indeed, these specific features of hLMS are related to *SRF/MYOCD*, the main drivers of smooth muscle cell differentiation, given that PTEN also interacts with SRF [70]. 

### 3.8. hLMS Can Be Targeted Specifically with an SRF/MYOCD Inhibitor

**Figure 6:**
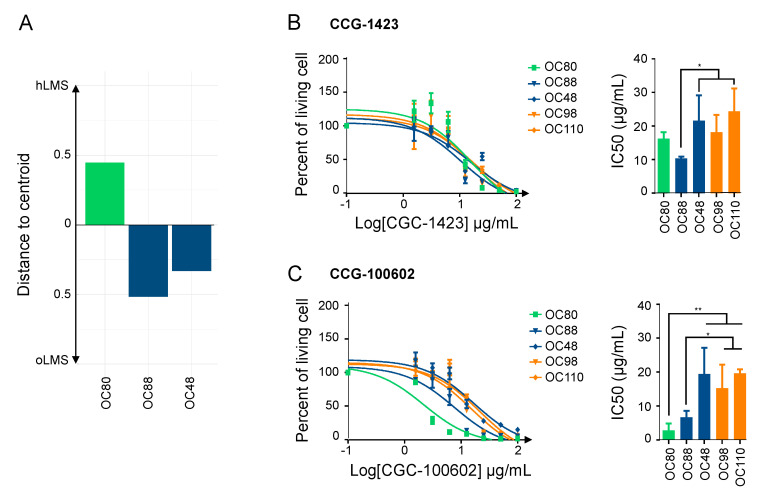
SRF/MYOCD inhibitors can specifically target hLMS. (**A**). Distance to centroids determining h/oLMS status on 1672 genes of 3 LMS cell lines. (**B**). Cytotoxicity curves of CCG-1423, inhibitor of SRF/MRTF axis, on 3 LMS and 2 UPS cell lines, using MTT assay after 72 h of treatment at increasing concentrations (from 1.5 to 100 µg/mL). The first graph represents one of the three experimentations used to determine IC50 with GraphPad. The second graph shows IC50 (mean ± s.d.; N = 3 independent assays). (**C**). Cytotoxicity curves of CCG-100206, an inhibitor of SRF/MYOCD axis, on same cell lines, using MTT assay after 72 h of treatment at same increasing concentrations. The first graph represents one of the three experimentations used to determine IC50 with GraphPad. The second graph shows IC50 (mean ± s.d.; N = 3 independent assays). * *p* ≤ 0.05, ** *p* ≤ 0.01, the *p*-value was calculated with unpaired *t*-test for (**B**,**C**).

We tested the hypothesis that the SRF/MYOCD axis could be a driver of hLMS oncogenesis by investigating the therapeutic inhibition of this pathway. We thus studied the impact on cell viability of inhibitors explicitly targeting the SRF/MYOCD pathway. CCG-1423, an inhibitor of the SRF/MRTF interaction [71], and CCG-100602, an inhibitor of the SRF/MYOCD interaction [72], were tested on 3 LMS (OC80: hLMS with an *MYOCD* amplification, OC48: oLMS with an *MYOCD* gain and OC88: oLMS) and 2 UPS (OC98 and OC110) cell lines (Figure 6A). 

After 72 h of treatment with increasing concentrations of each CCG, the cell viability assay showed that all cell lines were sensitive to both inhibitors (IC50 ranging from 2.56 ± 1.36 µg/mL to 21.41 ± 3.95 µg/mL) (Figure 6B,C). Regardless of their subgroup, LMS was slightly more sensitive to CCG-1423 than UPS, with OC88 reaching significance and being more affected than OC110 (Figure 6B). Interestingly, responses to CCG-100602, which is specific to the SRF/MYOCD interaction, exhibited three kinds of behavior: OC80 (hLMS; IC50 = 2.85 ± 1.15 µg/mL) was the most sensitive, OC88 (oLMS; IC50 = 6.70 ± 0.95 µg/mL) had an intermediate response, and OC48 (oLMS; IC50 = 19.44 ± 3.88 µg/mL) and the two UPS (IC50 = 15.32 ± 3.42 and 19.69 ± 0.67 µg/mL) were the least receptive (Figure 6C). Overall, all cell lines had a lower IC50 with the SRF/MYOCD inhibitor than with the SRF/MRTF inhibitor. However, the higher responsiveness of hLMS compared to others when SRF/MYOCD was inhibited indicates that the oncogenic dependency on the SRF/MYOCD axis is stronger in hLMS.

## 4. Discussion

Leiomyosarcoma is a very aggressive disease, despite its differentiation status, which remains poorly understood. Although previous studies highlighted molecular subgroups and showed the importance of stratifying patients to propose targeted and more effective therapies, no consistent biological or oncogenic mechanisms have yet emerged [3,4,5,6,7,8,9,10,17]. 

By integrating large-scale transcriptomic, epigenetic, and genomic data from an extensive series of LMS, we identified two groups of LMS that are barely similar to the transcriptomic subtypes previously described. Our study differs from the others as we analyzed primary tumors and excluded uterine LMS, unlike most other authors [3,6,8,9]. Also, the clinical characteristics of each group were similar across the three cohorts, strengthening the signature determining these two LMS sub-groups. hLMS are highly differentiated, preferentially carried by females, low-grade, and with an intra-abdominal location, while oLMS are poorly differentiated, high-grade, and located in the extremities. Both groups relapsed in at least 50% of cases in all three cohorts, and we observed a significantly poorer relapse-free survival of hLMS in the Affymetrix cohort. The lack of statistical significance and power in the ICGC and TCGA cohorts may be due to unbalanced group sizes and under-representation of oLMS cases, in contrast with the Affymetrix cohort. 

At the molecular level, transcriptomic profiling of oLMS tumors for both mRNA and miRNA highlighted variability within this group, and their genomic alteration patterns were not concordant between the cohorts. Moreover, oLMS showed many regulatory and functional features (active histone marks, many TF binding sites predicted from a cell line showing an epithelial morphology and epithelial-to-mesenchymal related terms) associated with fibroblasts, adipocytes, mesenchymal stem cells (MSC) and epithelial cells. They were spread among normal lung, adipose, and breast tissues in the GTEX analysis. Therefore, they could derive (i) from the de-differentiation of cells at their location; (ii) directly from circulating or local MSC; (iii) from the fusion between circulating or local MSC with a cell from the tumor site [73]. Yadav et al. [74] showed that cancer cells activate an unfolded protein response (UPR) to adapt to the external (undifferentiated phenotype in a specialized environment) or internal (managing extra material after fusion) micro-environment. Regardless of the origin of oLMS, this mechanism could explain the over-representation of UPR functional terms associated with oLMS over-expressed genes. The heterogeneity observed among patients with oLMS and the difficulty in defining unique oncogenesis might be due to their heterogeneous cellular origin.

In contrast, hLMS seem to have a unique cellular origin closely related to vascular smooth muscles. Indeed, when comparing hLMS with normal tissues, they homogeneously clustered with blood vessel samples. Moreover, over-expressed genes in hLMS, enriched in smooth muscle contractile functions, are mainly associated with active histone marks from smooth muscle datasets. Their promoters were enriched in predicted SRF binding sites (in silico and ChIP-seq), which are known to regulate the expression of targeted genes by binding to an element known as the CArG-box, located upstream of smooth muscle (SM) contractile genes [75,76]. To enhance SM differentiation, SRF must cooperate with the MYOCD protein [77], which was over-expressed in more than 84% of hLMS following genetic amplification in 36% of cases. Our data suggest that the over-expression of smooth muscle-related genes in hLMS (compared to oLMS) is triggered by MYOCD, as previously demonstrated by Pérot et al. [10]. Consistently, Dp427, a DMD isoform under the control of MYOCD [78], is nearly always expressed on the membrane in hLMS regardless of *DMD* genomic status, unlike in oLMS, in which it is no longer expressed. 

Interestingly, the SRF/MYOCD complex regulates all over-expressed miRNAs in hLMS, either directly upon binding to the CArG-boxes present in their promoter [65,79] or indirectly by targeting their host gene, as in *MIR28* and *LPP*, which has been shown to promote migration in differentiated LMS [10]. More than half of hLMS patients suffer a metastatic relapse. MIR143/145, which are over-expressed in hLMS, are integral components of the SRF/MYOCD network and have been shown to control stress fiber organization and to enhance migratory activity, thereby allowing the cytoskeletal remodeling and phenotypic switching of SMC during vascular injury [69]. MIR143/145 create complex feedback loops by repressing the expression of actin dynamics regulators and SRF/MRTF activity. Xin and colleagues suggested that the absence of these miRNAs creates an imbalance and that they are required for SMC cell migration. Additionally, Jiang et al. [65] showed that in differentiated human aortic vSMC, MIR1, regulated by SRF/MYOCD and overexpressed in hLMS, suppresses the expression of contractile proteins such as α-SMA and SM22, impairing actin organization, thus creating a negative feedback loop. MIR143/145 and MIR1 may help to fine-tune cytoskeletal homeostasis and allow migration of hLMS tumor cells.

hLMS are not only contractile and differentiated (contractile phenotype) but are also proliferative, with migratory features (synthetic phenotype) revealing the co-existence of both phenotypes. These characteristics are those of vSMC, which have highly plastic phenotypes and can cover a broad spectrum of phenotypes from synthetic to contractile [80]. Indeed, markers of both phenotypes, which are highly expressed in blood vessels, are among the most expressed in hLMS. This could represent the natural mixture of vSMC, spanning the phenotypic continuum in these cells. The synthetic phenotype is sustained by the propensity of hLMS to proliferate via strong enhancement of the cell cycle, as suggested by persistent *RB1* alterations, the significant over-expression of *E2F1*, the enrichment of up-regulated hLMS genes in E2F/RB1 targets, E2F7 binding sites and cell cycle functional related terms specific to hLMS. This contradicts the suggestion of Hemming et al. [8], who reported that all LMS possess this feature. It is probably due to the low number of “other” LMS that they had in the different cohorts when comparing LMS with other STS, thus highlighting what they considered to be their “conventional” characteristics. 

Although acquired through different preferential mechanisms, alteration of the tumor suppressors *TP53*, *RB1*, and *PTEN* occurred equally in hLMS and oLMS. It may boost proliferation and cell survival in a non-specific manner. However, the PTEN protein involved in SMC differentiation through direct interaction with SRF Field [70] is absent in hLMS. In fact, the SRF-regulating abilities of SMC are dual: they control the expression of both smooth muscle (SM) contractile genes, depending on MYOCD, and growth-related immediate early genes (IEG) [75], depending on ELK1. Horita and colleagues showed that in the nucleus, PTEN is directly linked to SRF and MYOCD to form a multi-protein complex and is essential for SRF binding to SM gene CArG-Boxes [70]. Upon forced SMC switch toward proliferation, PTEN is translocated into the cytoplasm, allowing the SRF-cofactor switch and increasing IEG gene expression. Therefore, the total absence of PTEN in hLMS may allow SRF to be linked with both SM genes, promoted by the large amount of MYOCD and IEG CArG-Boxes, so that SRF may express SMC contractile and proliferative genes. *MYOCD* amplification may overcome PTEN loss and lead to a significantly lower expression of ETS-family target genes in hLMS than in oLMS. Nevertheless, it should be remembered that by comparing two tumor types, the activity of ETS-related transcription in hLMS may be underestimated. Moreover, the proliferative phenotype of hLMS may be promoted by miRNA repression. Indeed, MIR455, MIR424, and MIR503 under-expression were associated with an increase in the pulmonary artery and bladder SMC proliferation by targeting FGF7 [67], CCND1 and CALU [81], and INSR [82], respectively. FGF7 and INSR are thus significantly over-expressed in hLMS and may participate in cell cycle enhancement. Finally, we observed global repression of the *DLK1-DIO3* miRNAs cluster, which is highly specific to hLMS and papillary thyroid carcinomas. Its predicted target genes are involved in vasculature development and cell migration in both diseases [83] and may play a role in this dual phenotype. However, this cluster involves around 50 miRNAs with different functions in different cellular contexts [84], so understanding how this repression impacts hLMS biology requires further investigation. 

## 5. Conclusions

Altogether, our findings show that hLMS may originate from vSMC, retain a remarkable degree of plasticity, grow in a maintained differentiated state, and benefit from the enhanced contractile apparatus to migrate. Our data show that the contractile abilities of hLMS come from their vSMC origin rather than from an acquired phenotype [10] and suggest that over-expression of *MYOCD* is positively selected, likely triggering tumorigenesis. Accordingly, functional inhibition assays with MYOCD/SRF inhibitors showed efficiency on hLMS, inducing cell death. 

## Figures and Tables

**Table 1:** Clinical enrichment and gene expression homogeneity in 42 hLMS vs. 56 oLMS from Affymetrix cohort and 73 hLMS vs. 29 oLMS in combined ICGC (28 vs. 11) and TCGA (45 vs. 18). (%) indicates that numbers in hLMS and oLMS columns are percentages of patients annotated with first feature (Well: well differentiated, Low: grade 1 + 2, F: female, Internal trunk). M: Male, other: Extremities, Trunk wall, limbs. The *p*-value was computed using Fisher’s Exact test. Otherwise, the median is reported, and the *p*-value was obtained with Wilcoxon’s test. (+) next to *p*-values indicates a significant enrichment in hLMS, while (−) indicates a significant enrichment in oLMS.

		Affymetrix			ICGC + TCGA		
Feature	Test	hLMS	oLMS	*p*-Value	hLMS	oLMS	*p*-Value
Differentiation (%)	Well (vs. Poor)	88	24	3.87 × 10^−9 (+)^	84	41	1.83 × 10^−5 (+)^
Grade (%)	Low (vs. High)	58	24	6.51 × 10^−4 (+)^			
Sex (%)	F (vs. M)	76	48	0.003 ^(+)^	68	41	0.007 ^(+)^
Location (%)	Internal trunk (vs. other)	60	7	8.48 × 10^−9 (+)^	82	27	1.53 × 10^−7 (+)^
Mitotic counts (median)	Ranks	17	24.5	0.009 ^(−)^	11	35	0.0006 ^(−)^
Gene expression variance (median)	Ranks	0.7	1	2.90 × 10^−13 (−)^	ICGC		
					0.25	0.36	<2.2 × 10^−16 (−)^
					TCGA		
					0.45	0.9	<2.2 × 10^−16 (−)^

**Table 2:** Summary of genetic alterations in 39 ICGC patients for *TP53*, *RB1*, *PTEN*, *ATRX* and *DMD*. Alterations are categorized as follows: mutation: missense, nonsense, frameshift (FS), non-FS, splicing, SV: structural variant, loss: loss of at least one allele, total: number of patients carrying at least one alteration. Numbers indicate the percentage of patients harboring the alteration; the actual numbers are reported between brackets.

Group	Alterations	*TP53*	*RB1*	*PTEN*	*ATRX*	*DMD*
hLMS	mutation	60.7 (17)	21.4 (6)	0 (0)	7.1 (2)	3.6 (2)
oLMS		18.2 (2)	9 (1)	0 (0)	27.3 (3)	9 (1)
all		48.7 (19)	17.9 (7)	0 (0)	12.8 (5)	5.1 (3)
hLMS	SV	25 (7)	35.7 (10)	3.6 (1)	7.1 (2)	14.3 (4)
oLMS		36.4 (4)	36.4 (4)	0 (0)	0(0)	36.4 (4)
all		28.2 (11)	35.9 (14)	2.6 (1)	5.1 (2)	20.5 (8)
hLMS	loss	89.3 (25)	92.9 (26)	82.1 (23)	7.1 (2)	3.6 (1)
oLMS		90.9 (10)	81.8 (9)	81.8 (9)	18.1 (2)	0 (0)
all		89.7 (35)	89.7 (35)	82 (32)	10.2 (4)	2.6 (1)
hLMS	total	100 (28)	100 (28)	82.1 (23)	21.4 (6)	17.8 (5)
oLMS		100 (11)	90.9 (10)	81.8 (9)	45.5 (5)	45.5 (5)
all		100 (39)	97.4 (38)	82 (32)	28.2 (11)	25.6 (10)

## Data Availability

Genomic and expression arrays for the other sample are available on Gene Expression Omnibus (GEO) under accession GSE159847 and GSE159848. ICGC cohort Whole-Genome sequencing and RNA sequencing data for the 67 LMS are available at https://dcc.icgc.org/projects/LMS-FR, accessed on 14 January 2023. miRNA data are available on Gene Expression Omnibus (GEO) under accession GSE159849. Raw files are available on Sequence Read Archive under accessions SRP288162. Correspondence between previously published sample identifiers in Gene Expression Omnibus (GSE40021, GSE21050, GSE23980, GSE71118, GSE154591) and in ArrayExpress (E-MTAB-373) datasets and identifiers used in this paper is presented in Table S1. The code is available at https://github.com/ElodieDarbo/lms_onco, accessed on 14 January 2023. Details for software availability and public datasets are in File S1.

## Supplementary Materials

The following supporting information can be downloaded at: https://www.mdpi.com/article/10.3390/cancers15020534/s1. Figure S1 related to methods and Figure 1A: Normalization of Affymetrix and Agilent microarrays. Figure S2 related to Figure 2: Heatmap showing median expression level per tissue type of most expressed hLMS 100 genes. Figure S3 pertaining to Figure 3: Evaluation of DIO3-DLK1 miRNA cluster expression. Figure S4 related to Figure 5: Copy number alterations and mutational burden. Table S1 related to Methods: Presentation of a cohort of 555 samples analyzed on micro-arrays and 84 Affymetrix cohort samples for which copy number data were analyzed. Table S2 related to Methods: Clinicopathological characteristics of patients from Affymetrix (98 patients), TCGA (75 patients), and ICGC (59 patients). Table S3 related to Figure 1 and Figure 3: Results of functional annotations and enrichment in co-expressed modules, differentially expressed genes in Affymetrix cohort, top hundred most expressed genes in hLMS, and miRNA target genes. Table S4 related to Figure 3: Table summarizing differential expression analysis for mRNA in Affymetrix, ICGC, and TCGA, for miRNA in ICGC and TCGA, and miRComb miRNA-mRNA interaction analysis. Table S5 related to Figure 4: Table summarizing copy number alteration analysis by cytogenic band and genes in the merged cohort. Table S6 related to Figure 5: (**A**). Table summarizing genetic alterations occurring for *TP53*, *RB1*, *PTEN*, *DMD*, *ATRX*, and *MYOCD* in each sample of ICGC cohort as well as expression data of the six genes. (**B**). A table presenting mutations detected in *TP53*, *RB1*, *DMD*, and *ATRX*. (**C**). A table presenting structural variants detected in *TP53*, *RB1*, *DMD*, *PTEN*, and *ATRX*. (**D**). Tables presenting primers used on genomic DNA to validate mutations and structural variants. (**E**). Table describing primers used on cDNA to validate mutations and fusion transcripts. File S1 related to methods provides details about DNA, RNA and miRNA extraction and sequencing, breakpoint detection strategy, cross micro-array platform normalization and a key resources table. References [10,11,29,30,31,50] are cited in File S1.

## Abbreviations

LMS	leiomyosacoma
hLMS	homogenous leiomyosarcoma
oLMS	other leiomyosarcoma
GIST	Gastrointestinal Stromal Tumor
PCC	Pearson’s Correlation coefficient
TF	transcription factor
ECM	extracellular matrix
DE	differentially expressed
vSMC	vascular smooth muscle cell
UPR	unfolded protein response
