# Supplementary material for: Distinct Cellular Origins and Differentiation Process Account for Distinct Oncogenic and Clinical Behaviors of Leiomyosarcomas
Source: Cancers (Basel). 2023 Jan 15;15(2):534. doi: 10.3390/cancers15020534 (PMC9856933; doi:10.3390/cancers15020534)
Supplement: Supplementary file 1 [file cancers-15-00534-s001.zip › File_S1.pdf]

Supplementary methods

RNA extraction

RNA extraction was performed using standard TRIzol (15596026, Thermo Fisher, Waltham, MA, USA) / chloroform extraction (32211-1L, Fisher Scientific, Hampton, NH, USA) followed by 100% ethanol precipitation and RNA purification using the RNeasy Mini Kit (74104, Qiagen, Hilden, Germany) with DNase treatment (79254, RNase-Free DNase Set, Qiagen, Hilden, Germany). Total RNA was quantified using a Nanodrop 1000 spectrophotometer (Thermo Scientific, Waltham, MA, USA) and qualified with an Agilent 2100 Bioanalyzer (Agilent, Santa Clara, CA, USA) using the Agilent RNA 6000 Nano Kit (5067-1511, Agilent, Santa Clara, CA, USA) according to the manufacturer's instructions.

RNA sequencing from frozen samples

To control the sequencing quality, ERCC RNA Spike-In Mix (4456740, Life technologies, Carlsbad, CA, USA) were added to each RNA sample as recommended by the manufacturer. Analysis of this quality control was performed as previously described [1].

Libraries from total RNA were prepared using the TruSeq®Stranded Total RNA Gold Library Preparation Kit (RS-122-2301, Illumina Inc., San Diego, CA, USA) according to the manufacturer's protocol. Briefly, 0.5 µg of total

RNA was ribo-depleted using the Ribo-Zero Gold Kit. RNA fragmentation resulted in fragments of 80 – 450 nt, with a major peak at 160 nt. First-strand cDNA synthesis by random hexamers and reverse transcriptase was followed by second-strand cDNA synthesis, performed in presence of dUTP instead of dTTP. Blunt-ended double-stranded cDNA was 3′adenylated and Illumina-indexed adapters were ligated. Resulting libraries were enriched with 15 PCR cycles.

Libraries were sequenced on HiSeq2000 (Illumina Inc., San Diego, CA, USA) in paired-end mode with a read length of 2x75 bp using TruSeq SBS Kit v3-HS (FC-401-3001, Illumina Inc., San Diego, CA, USA). Image analysis, base calling and base quality scoring of the run were processed by integrated primary Real Time Analysis (RTA 1.13.48, Illumina Inc., San Diego, CA, USA) software and followed by generation of FASTQ sequence files by CASAVA (v1.8, Illumina Inc., San Diego, CA, USA). Library construction and RNA sequencing were performed at the Centro Nacional de Análisis Genómico (CNAG, Barcelona, Spain).

miRNA sequencing

Libraries were prepared by the Genomics Unit at the Centre de Regulació Genòmica (Barcelona, Spain) using the NEBNext Small RNA Library Prep Set for Illumina (E7330L, New England Biolabs Inc., Ipswich, MA, USA) according to the manufacturer's recommendations. Libraries were sequenced

on HiSeq2500 (Illumina Inc., San Diego, CA, USA) in single-read mode with a read length of 50bp (V4 chemistry) to reach a minimal yield of 10M reads.

DNA extraction

Genomic DNA from frozen samples of the ICGC cohort was isolated using standard phenol-chloroform extraction protocol. DNA was quantified using Nanodrop 1000 spectrophotometer (Thermo Fisher Scientific, Waltham, MA, USA). Genomic DNA from blood samples was extracted using customized automated purification of DNA from compromised blood samples on the Autopure LS device according to the manufacturer's recommendations (9001340, Qiagen, Hilden, Germany) with increased centrifugation of 10 min for DNA precipitation and DNA wash.

Whole genome sequencing

Whole genome sequencing was performed only on the ICGC cohort. To construct short-insert paired-end libraries, a no-PCR protocol was used with the TruSeq™DNA Sample Preparation Kit v2 (FC-121-2001/FC-121-2002, Illumina Inc., San Diego, CA, USA) and the KAPA Library Preparation Kit (KK8235, Kapa Biosystems, Basel, Switzerland). Briefly, 2 μg of genomic DNA were sheared on a Covaris™ E220, size-selected and concentrated using AMPure XP beads (A63880, Agencourt, Beckman Coulter, Brea, CA, USA) in order to reach a fragment size of 220 – 480 bp. Fragmented DNA was end-

repaired, adenylated and ligated to Illumina-specific indexed paired-end adapters.

DNA sequencing was performed in paired-end mode, 2x100 bp or 2x125 bp according to flowcell version, in five or three sequencing lanes of HiSeq2000 flowcell v3 or v4 (Illumina Inc., San Diego, CA, USA) to analyze tumor or normal/constitutive samples and to reach minimal yield of 145 or 85 Gb, respectively. Two tumor samples were sequenced in 20 lanes to reach a minimal yield of 560 Gb. Image analysis, base calling and quality scoring of the run were processed using the manufacturer's Real Time Analysis software (RTA 1.13.48) and followed by generation of FASTQ sequence files by CASAVA (Illumina Inc., San Diego, CA, USA).

Breakpoints detection

The algorithm followed three main steps as detailed bellow.

i) Identification: at this step, reads with at least one soft-clipped end were analyzed as singletons. A position was considered as a potential breakpoint if it was covered by at least 4 soft-clipped reads, 5 soft-clipped bases (with at least two occurrences of two different bases), and if they represented more than 5% of the total amount of reads at this position in the tumor sample. We selected potential somatic events by discarding positions covered by at least 1 read and 1 base in a surrounding 5-nucleotide window in the normal sample. We refer to them as the "first side" of the breakpoint. ii) Characterization: to

determine the genomic positions of the soft-clipped sequence from selected reads, we used the UCSC blat server [2]. If no match was returned, the reverse complement sequence was pulled to test. If there was still no match, the BAM file was investigated for some soft-clip somatic position around the discordant or oversized-insert read mate (hereafter named abnormal) location from the first side of the breakpoint. Because of the small size of the soft-clipped sequence, multiple matches can be found. We used soft-clipped abnormal read mates to select matches with the most coherent chromosomic locations. We refer to them as the “second side” of the breakpoint. iii) Selection: Positions detected from both the first and second sides (in a 5-nucleotide window) were defined as the common pool. We considered as artifacts (due to repeat regions for instance) couples of positions covered with reads and associated soft-clipped sequences separated by fewer than 15 nucleotides and discarded them. We classified the breakpoints in three groups: high-confidence breakpoints, breakpoints needing investigation, and unique position breakpoints. If a breakpoint was covered by reads and associated soft-clipped sequences having both positions belonging to the common pool, it was classified in the first group. If a breakpoint was covered by reads and associated soft-clipped sequences having only one of the positions belonging to the common pool, it was classified in the second group. Then the missing position was searched among the filtered positions. If it was present in the normal sample, the position was discarded and the

breakpoint was completed otherwise. Finally, the third group corresponds to breakpoints with both sides outside the common pool and considered as unique: these were discarded. The sides of breakpoints were sorted according to their chromosomic positions to avoid duplicates.

Normalization of Affymetrix and Agilent micro-arrays and gene selection

Selecting consistent genes between Affymetrix and Agilent:

$cor_{max,i} =$

$max\left(cor\left(g_{Affy,i}, g_{Agi,\forall j\neq i}\right), cor\left(g_{Affy,i}, g_{Affy,\forall j\neq i}\right), cor\left(g_{Agi,i}, g_{Agi,\forall j\neq i}\right)\right)$

$g_i = \left\{1\ Cor\left(g_i, g_{j=i}\right) > 0.8 \lor cor\left(g_i, g_{j=i}\right) > cor_{max,i} \,\middle|\, 0\ otherwise\right\}$

Where $i, j \in \mathcal{A}\{1..n\}$ are the indexes of pairs of genes to be compared. g_{Affy} and g_{Agi} are genes analyzed on Affymetrix and Agilent micro-arrays and $\mathcal{A}$ the ensemble of all possible indexes between 1 and n the number of genes to be tested. g_i represents the status of gene i that will be kept if 1 or discarded otherwise.

Median harmonization:

$median_{i,Affy} = median\left(g_{i,\forall k\in\{Affy\}}\right)$

$median_{i,Agi} = median\left(g_{i,\forall k\in\{Agi\}}\right)$

$median_{i,All} = mean(median_{i,Affy}, median_{i,Agi})$

$g_{i,AffyHarm} = g_{i,Affy} - median_{i,Affy} + median_{i,All}$

$g_{i,AgiHarm} = g_{i,Agi} - median_{i,Agi} + median_{i,All}$

Where $i \in \{1..n\}$ is the gene index and n the number of genes to be analyzed, g represents the gene expression either in Affymetrix samples ($Affy$) or Agilent samples (Agi).

Visualization

All generated plots were produced using ggplot2 R package version 3.3.0 [3] and extension ggpubr version 0.2.5 [4] unless explicitly said otherwise.

KEY RESOURCES TABLE

REAGENT or RESOURCE	SOURCE	IDENTIFIER
Antibodies		
Rabbit monoclonal anti-PTEN (Clone 138G6)	Cell Signaling Technology	Cat# 9559, RRID:AB_390810
Mouse monoclonal anti-Human Dystrophin (Clone Dy4/6D3	Leica Biosystems	Cat# NCL-DYS1, RRID:AB_442080
Rabbit polyclonal anti-Dystrophin	Abcam	Cat# ab15277, RRID:AB_301813
Goat anti-Mouse IgG (H+L) Cross-Adsorbed Secondary Antibody, Alexa Fluor 488	Thermo Fisher Scientific	Cat# A-11001, RRID:AB_2534069
F(ab')2-Goat anti-Rabbit IgG (H+L) Cross-Adsorbed Secondary Antibody, Alexa Fluor 594	Thermo Fisher Scientific	Cat# A-11072, RRID:AB_2534116
Biological Samples		
GIST (60 samples)	Bergonié Institute	[5]
Synovial sarcoma (58 samples)	Bergonié Institute	[6]
Complex sarcomas (278 samples)	Bergonié Institute	[7]<br>[8,9]<br>[1]
Complex sarcomas (159 samples)	Bergonié Institute	This paper
ICGC cohort of human LMS (frozen sample, FFPE blocs, clinical data)	French Sarcoma Group as part of the ICGC program	This paper
Chemicals, Peptides, and Recombinant Proteins		
CCG-1423	Bertin Bioreagent	Cat# 10010350, CAS:285986-88-1
CCG-100602	Bertin Bioreagent	Cat# 10787, CAS:1207113-88-9
Critical Commercial Assays		
High Capacity cDNA	Applied Biosystems	Cat# 4368814

Reverse Transcription Kit		
AmpliTaqGold® DNA polymerase	Applied Biosystems	Cat# 4311820
TruSeq™DNA Sample Preparation Kit v2	Illumina	Cat# FC-121-2001/FC-121-2002
ERCC RNA Spike-In Mix	Life Technologies	Cat# 4456740
TruSeq®Stranded Total RNA Gold Library Preparation Kit	Illumina	Cat# RS-122-2301
NEBNext Small RNA Library Prep Set for Illumina	New England Biolabs Inc.	Cat# E7330L
MYOCD FISH probe	Empire Genomics	Cat# EG-MYOCD-CHR17-20ORGR
Deposited Data		
Whole-Genome sequencing and RNA sequencing ICGC cohort	This paper	https://dcc.icgc.org/projects/LMS-FR
miRNA sequencing data ICGC cohort	This paper<br>Availability on 2022/06/30	Sequence Read Archive: SRP288162<br>Gene Expression Omnibus: GSE159849
Agilent micro-array Complex sarcomas	This paper<br>Availability on 2022/06/30	Gene Expression Omnibus: GSE159847
Agilent micro-array myxoid LPS	This paper<br>Availability on 2022/06/30	Gene Expression Omnibus: GSE159848
Agilent micro-array GIST	[5]	ArrayExpress: E-MTAB-373
Agilent micro-array Synovial sarcoma	[6]	Gene Expression Omnibus: GSE40021
Affymetrix micro-array Complex sarcomas	[7]<br>[8,9]<br>[1]	Gene Expression Omnibus: GSE21050, GSE23980, GSE71118
RNA-seq expression (log2+1 RSEM normalised) (TCGA SARC: version 2015-02-24)	[10]	RRID:SCR_018938<br>http://xena.ucsc.edu/
miRNA expression (TCGA SARC: version 2017-09-08, PANCAN batch corrected: version 2016-12-29)	[10]	RRID:SCR_018938<br>http://xena.ucsc.edu/
Copy Number Variant (gene level GISTIC2) (TCGA SARC: version 2017-09-08)	[10]	RRID:SCR_018938<br>http://xena.ucsc.edu/
GTEX+TCGA combined expression data (version 2016-04-12)	[10]	RRID:SCR_018938<br>http://xena.ucsc.edu/
Clinical annotations (TCGA SARC)	[11]	https://genome-cancer.ucsc.edu/

SNP6 arrays	[12]	Gene Expression Omnibus : GSE154591
miRBase (Downloaded Feb 2020)	[13]	http://www.mirbase.org/
miRecords version 4	[14]	RRID:SCR_013021 http://c1.accurascience.com/miRecords/
miRTarBase version 7.0	[15]	RRID:SCR_017355 http://mirtarbase.mbc.nctu.edu.tw/
Catalogue Of Somatic Mutations In Cancer (COSMIC) signatures version 3.1	[16]	RRID:SCR_002260 https://cancer.sanger.ac.uk/cosmic/
Experimental Models: Cell Lines		
OC80	This paper	NA
OC88	This paper	NA
OC48	This paper	NA
OC98	This paper	NA
OC110	This paper	NA
Oligonucleotides		
See supplementary Table S4D and S4E	This paper	NA
Software and Algorithms		
Illumina Real Time Analysis	Illumina	RRID:SCR_014332
CASAVA version 1.8	Illumina	RRID:SCR_001802 http://support.illumina.com/sequencing/sequencing_software/casava.html
Sickle2	[17]	RRID:SCR_006800 https://github.com/najoshi/sickle
SeqPrep3		RRID:SCR_013004 https://github.com/jstjohn/SeqPrep
Bowtie version 2.2.1.0	[18]	RRID:SCR_005476 http://bowtie-bio.sourceforge.net/index.shtml
SAMtools version 0.1.19, 1.9	[19]	RRID:SCR_002105 http://htslib.org/
bcfTools version 0.2.0	[20]	https://samtools.github.io/bcftools/
PicardTools version 1.118	[21]	RRID:SCR_006525 http://broadinstitute.github.io/picard/
Defuse version 0.6.1	[22]	RRID:SCR_003279 https://bitbucket.org/dranew/defuse/src/master/
Cutadapt version 1.10	[23]	RRID:SCR_011841 https://cutadapt.readthedocs.io/

		en/stable/
FastQC	Babraham Institue	RRID:SCR_014583 http://www.bioinformatics.babraham.ac.uk/projects/fastqc/
BWA-aln version 0.7.17	[24]	RRID:SCR_010910 http://bio-bwa.sourceforge.net/bwa.shtml
Qualimap version 2.2.2b	[25]	http://qualimap.conesalab.org/
Annovar version 20160314	[26]	RRID:SCR_012821 http://www.openbioinformatics.org/annovar/
Integrative Genomics Viewer (IGV) version 2.6.3	[27]	RRID:SCR_011793 http://www.broadinstitute.org/igv/
Primer 3 version 0.4.0	[28]	RRID:SCR_003139 https://bioinfo.ut.ee/primer3-0.4.0/
FinchTV version 1.4.0	Geospiza	RRID:SCR_005584 http://www.geospiza.com/Products/finchtv.shtml
Fiji	[29]	RRID:SCR_002285 http://fiji.sc
GraphPad Prism	GraphPad	RRID:SCR_002798 http://www.graphpad.com/
Gene Set Enrichment Analysis (GSEA)	[30]	RRID:SCR_003199 http://www.broadinstitute.org/gsea/
BLAT	[31]	RRID:SCR_011919 http://genome.ucsc.edu/cgi-bin/hgBlat?command=start
TxDb.Hsapiens.UCSC.hg19.knownGene version 3.2.2	[32], R package	http://bioconductor.org/packages/release/data/annotation/html/TxDb.Hsapiens.UCSC.hg19.knownGene.html
edgeR version 3.28.1	[33], R package	RRID:SCR_012802 http://bioconductor.org/packages/edgeR/
miRComb	[34], R package	https://github.com/mariavica/mircomb
ggplot2 version 3.3.0	[3], R package	RRID:SCR_014601 https://cran.r-project.org/web/packages/ggplot2/index.html
ggpubr version 0.2.5	[4], R package	https://CRAN.R-project.org/package=ggpubr
ggbiplot	[35], R package	https://github.com/vqv/ggbiplot
clusterProfiler version 3.14.3	[36], R package	RRID:SCR_016884 http://bioconductor.org/packages/release/bioc/html/clusterProfiler.html

FactoMineR version 2.3	[37], R package	RRID:SCR_014602 http://factominer.free.fr/index.html
preprocessCore version 1.48.0	[38], R package	https://bioconductor.org/packages/release/bioc/html/preprocessCore.html
pheatmap version 1.0.12	[39], R package	RRID:SCR_016418 https://www.rdocumentation.org/packages/pheatmap/versions/0.2/topics/pheatmap
Rtsne version 0.15	[40], R package	RRID:SCR_016342 https://cran.r-project.org/web/packages/Rtsne/index.html
citccmst version 1.0.2	[41], R package	https://CRAN.R-project.org/package=citccmst
cn.MOPS	[42], R package	RRID:SCR_013036 http://bioconductor.org/packages/2.12/bioc/html/cn.mops.html
GSAn	[43], online tool	https://gsan.labri.fr/
iCistarget	[44], online tool	https://gbiomed.kuleuven.be/apps/lcb/i-cisTarget/
igraph version 1.2.5	[45], R package	RRID:SCR_019225 https://igraph.org/r/
mclust version 5.4.6	[46], R package	https://CRAN.R-project.org/package=mclust
MutationalPatterns version 1.12.0	[47], R package	https://bioconductor.org/packages/release/bioc/html/MutationalPatterns.html
survival version 3.1-12	[48], R package	https://CRAN.R-project.org/package=survival
survminer version 0.4.6	[49], R package	https://CRAN.R-project.org/package=survminer
R version 3.6	R Project for Statistical Computing	RRID:SCR_001905 http://www.r-project.org/
Molecular Signatures Database (MSigDB) version 6	[50], Broad institute	RRID:SCR_016863 http://software.broadinstitute.org/gsea/msigdb/index.jsp
Analytic pipeline	This paper	OCEANCODE: provisional DOI:10.24433/CO.0299110.v1 https://github.com/ElodieDarbo/lms_onco

References

"Gene set enrichment analysis: a knowledge-based approach for interpreting genome-wide expression profiles" has comments on PubPeer

1. Lesluyes, T.; Pérot, G.; Largeau, M.R.; Brulard, C.; Lagarde, P.; Dapremont, V.; Lucchesi, C.; Neuville, A.; Terrier, P.; Vince-Ranchère, D.; et al. RNA sequencing validation of the Complexity INdex in SARComas prognostic signature. *Eur. J. Cancer* **2016**, *57*, 104–111, https://doi.org/10.1016/j.ejca.2015.12.027.
2. Kent, W.J. BLAT—The BLAST-Like Alignment Tool. *Genome Res.* **2002**, *12*, 656–664, https://doi.org/10.1101/gr.229202.
3. Wickham, H. *ggplot2: Elegant Graphics for Data Analysis*; Springer-Verlag: New York, NY, USA, 2016; ISBN 978-3-319-24277-4. Available online: https://ggplot2.tidyverse.org (accessed on).
4. Kassambara, A. Ggpubr: "ggplot2" Based Publication Ready Plots 2020.
5. Lagarde, P.; Pérot, G.; Kauffmann, A.; Brulard, C.; Dapremont, V.; Hostein, I.; Neuville, A.; Wozniak, A.; Sciot, R.; Schöffski, P.; et al. Mitotic Checkpoints and Chromosome Instability Are Strong Predictors of Clinical Outcome in Gastrointestinal Stromal Tumors. *Clin. Cancer Res.* **2012**, *18*, 826–838, https://doi.org/10.1158/1078-0432.ccr-11-1610.
6. Lagarde, P.; Przybyl, J.; Brulard, C.; Pérot, G.; Pierron, G.; Delattre, O.; Sciot, R.; Wozniak, A.; Schöffski, P.; Terrier, P.; et al. Chromosome Instability Accounts for Reverse Metastatic Outcomes of Pediatric and Adult Synovial Sarcomas. *J. Clin. Oncol.* **2013**, *31*, 608–615, https://doi.org/10.1200/jco.2012.46.0147.
7. Chibon, F.; Lagarde, P.; Salas, S.; Pérot, G.; Brouste, V.; Tirode, F.; Lucchesi, C.; de Reynies, A.; Kauffmann, A.; Bui, B.; et al. Validated prediction of clinical outcome in sarcomas and multiple types of cancer on the basis of a gene expression signature related to genome complexity. *Nat. Med.* **2010**, *16*, 781–787, https://doi.org/10.1038/nm.2174.
8. Gibault, L.; Pérot, G.; Chibon, F.; Bonnin, S.; Lagarde, P.; Terrier, P.; Coindre, J.-M.; Aurias, A. New insights in sarcoma oncogenesis: a comprehensive analysis of a large series of 160 soft tissue sarcomas with complex genomics. *J. Pathol.* **2010**, *223*, 64–71, https://doi.org/10.1002/path.2787.
9. Gibault, L.; Ferreira, C.; Pérot, G.; Audebourg, A.; Chibon, F.; Bonnin, S.; Lagarde, P.; Vacher-Lavenu, M.-C.; Terrier, P.; Coindre, J.-M.; et al. From PTEN loss of expression to RICTOR role in smooth muscle differentiation: complex involvement of the mTOR pathway in leiomyosarcomas and pleomorphic sarcomas. *Mod. Pathol.* **2012**, *25*, 197–211, https://doi.org/10.1038/modpathol.2011.163.
10. Goldman, M.J.; Craft, B.; Hastie, M.; Repečka, K.; McDade, F.; Kamath, A.; Banerjee, A.; Luo, Y.; Rogers, D.; Brooks, A.N.; et al. Visualizing and interpreting cancer genomics data via the Xena platform. *Nat. Biotechnol.* **2020**, *38*, 675–678, https://doi.org/10.1038/s41587-020-0546-8.
11. Goldman, M.; Craft, B.; Swatloski, T.; Cline, M.S.; Morozova, O.; Diekhans, M.; Haussler, D.; Zhu, J. The UCSC Cancer Genomics Browser: update 2015. *Nucleic Acids Res.* **2014**, *43*, D812–D817, https://doi.org/10.1093/nar/gku1073.
12. Lesluyes, T.; Baud, J.; Pérot, G.; Charon-Barra, C.; You, A.; Valo, I.; Bazille, C.; Mishellany, F.; Leroux, A.; Renard-Oldrini, S.; et al. Genomic and transcriptomic comparison of post-radiation versus sporadic sarcomas. *Mod. Pathol.* **2019**, *32*, 1786–1794, https://doi.org/10.1038/s41379-019-0300-2.
13. Kozomara, A.; Griffiths-Jones, S. miRBase: integrating microRNA annotation and deep-sequencing data. *Nucleic Acids Res.* **2010**, *39*, D152–D157, https://doi.org/10.1093/nar/gkq1027.
14. Xiao, F.; Zuo, Z.; Cai, G.; Kang, S.; Gao, X.; Li, T. miRecords: an integrated resource for microRNA-target interactions. *Nucleic Acids Res.* **2008**, *37*, D105–D110, https://doi.org/10.1093/nar/gkn851.
15. Chou, C.-H.; Shrestha, S.; Yang, C.-D.; Chang, N.-W.; Lin, Y.-L.; Liao, K.-W.; Huang, W.-C.; Sun, T.-H.; Tu, S.-J.; Lee, W.-H.; et al. miRTarBase update 2018: a resource for experimentally validated microRNA-target interactions. *Nucleic Acids Res.* **2017**, *46*, D296–D302, https://doi.org/10.1093/nar/gkx1067.
16. Tate, J.G.; Bamford, S.; Jubb, H.C.; Sondka, Z.; Beare, D.M.; Bindal, N.; Boutselakis, H.; Cole, C.G.; Creatore, C.; Dawson, E.; et al. COSMIC: The Catalogue of Somatic Mutations in Cancer. *Nucleic Acids Res.* **2019**, *47*, D941–D947, https://doi.org/10.1093/nar/gky1015.
17. Joshi, N.; Fass, J. Sickle: A Sliding-Window, Adaptive, Quality-Based Trimming Tool for FastQ Files (Version 1.33) [Software]; 2011;
18. Langmead, B.; Salzberg, S.L. Fast gapped-read alignment with Bowtie 2. *Nat. Methods* **2012**, *9*, 357–359, https://doi.org/10.1038/nmeth.1923.
19. Li, H.; Handsaker, B.; Wysoker, A.; Fennell, T.; Ruan, J.; Homer, N.; Marth, G.; Abecasis, G.; Durbin, R. The Sequence Alignment/Map format and SAMtools. *Bioinformatics* **2009**, *25*, 2078–2079, https://doi.org/10.1093/bioinformatics/btp352.

20. Li, H. A statistical framework for SNP calling, mutation discovery, association mapping and population genetical parameter estimation from sequencing data. *Bioinformatics* **2011**, *27*, 2987–2993, doi:10.1093/bioinformatics/btr509.
21. Broad Institute Picard Toolkit; Broad Institute, GitHub repository, 2019;
22. McPherson, A.; Hormozdiari, F.; Zayed, A.; Giuliany, R.; Ha, G.; Sun, M.G.F.; Griffith, M.; Moussavi, A.H.; Senz, J.; Melnyk, N.; et al. deFuse: An Algorithm for Gene Fusion Discovery in Tumor RNA-Seq Data. *PLOS Comput. Biol.* **2011**, *7*, e1001138, https://doi.org/10.1371/journal.pcbi.1001138.
23. Martin, M. Cutadapt removes adapter sequences from high-throughput sequencing reads. *EMBnet. J.* **2011**, *17*, 10–12, doi:10.14806/ej.17.1.200.
24. Li, H.; Durbin, R. Fast and accurate short read alignment with Burrows—Wheeler transform. *Bioinformatics* **2009**, *25*, 1754–1760, https://doi.org/10.1093/bioinformatics/btp324.
25. García-Alcalde, F.; Okonechnikov, K.; Carbonell, J.; Cruz, L.M.; Götz, S.; Tarazona, S.; Dopazo, J.; Meyer, T.F.; Conesa, A. Qualimap: evaluating next-generation sequencing alignment data. *Bioinformatics* **2012**, *28*, 2678–2679, https://doi.org/10.1093/bioinformatics/bts503.
26. Wang, K.; Li, M.; Hakonarson, H. ANNOVAR: Functional annotation of genetic variants from high-throughput sequencing data. *Nucleic Acids Res.* **2010**, *38*, e164.
27. Robinson, J.T.; Thorvaldsdóttir, H.; Winckler, W.; Guttman, M.; Lander, E.S.; Getz, G.; Mesirov, J.P. Integrative genomics viewer. *Nat. Biotechnol.* **2011**, *29*, 24–26, https://doi.org/10.1038/nbt.1754.
28. Untergasser, A.; Cutcutache, I.; Koressaar, T.; Ye, J.; Faircloth, B.C.; Remm, M.; Rozen, S.G. Primer3—New capabilities and interfaces. *Nucleic Acids Res.* **2012**, *40*, e115, doi:10.1093/nar/gks596.
29. Schindelin, J.; Arganda-Carreras, I.; Frise, E.; Kaynig, V.; Longair, M.; Pietzsch, T.; Preibisch, S.; Rueden, C.; Saalfeld, S.; Schmid, B.; et al. Fiji: An open-source platform for biological-image analysis. *Nat. Methods* **2012**, *9*, 676–682, https://doi.org/10.1038/nmeth.2019.
30. Subramanian, A.; Tamayo, P.; Mootha, V.K.; Mukherjee, S.; Ebert, B.L.; Gillette, M.A.; Paulovich, A.; Pomeroy, S.L.; Golub, T.R.; Lander, E.S.; et al. Gene set enrichment analysis: A knowledge-based approach for interpreting genome-wide expression profiles. *Proc. Natl. Acad. Sci. USA* **2005**, *102*, 15545–15550. https://doi.org/10.1073/pnas.0506580102.
31. Kent, W.J.; Sugnet, C.W.; Furey, T.S.; Roskin, K.M.; Pringle, T.H.; Zahler, A.M.; Haussler, D. The Human Genome Browser at UCSC. *Genome Res.* **2002**, *12*, 996–1006, doi:10.1101/gr.229102.
32. Carlson, M. TxDb.Hsapiens.UCSC.Hg19.KnownGene: Annotation Package for TxDb Object(s 2015.
33. Robinson, M.D.; McCarthy, D.J.; Smyth, G.K. EdgeR: A Bioconductor package for differential expression analysis of digital gene expression data. *Bioinformatics* **2010**, *26*, 139–140, https://doi.org/10.1093/bioinformatics/btp616.
34. Vila-Casadesús, M.; Gironella, M.; Lozano, J.J. MiRComb: An R Package to Analyse miRNA-mRNA Interactions. Examples across Five Digestive Cancers. *PLOS ONE* **2016**, *11*, e0151127, https://doi.org/10.1371/journal.pone.0151127.
35. Vu, V.Q. Ggbiplot: A Ggplot2 Based Biplot 2011.
36. Yu, G.; Wang, L.-G.; Han, Y.; He, Q.-Y. clusterProfiler: An R Package for Comparing Biological Themes Among Gene Clusters. *OMICS J. Integr. Biol.* **2012**, *16*, 284–287, doi:10.1089/omi.2011.0118.
37. Lê, S.; Josse, J.; Husson, F. FactoMineR: An*R*Package for Multivariate Analysis. *J. Stat. Softw.* **2008**, *25*, 1–18, https://doi.org/10.18637/jss.v025.i01.
38. Bolstad, B. PreprocessCore: A Collection of Pre-Processing Functions 2019.
39. Kolde, R. Pheatmap: Pretty Heatmaps 2019.
40. van der Maaten, L.; Hinton, G. Visualizing non-metric similarities in multiple maps. *Mach. Learn.* **2011**, *87*, 33–55, https://doi.org/10.1007/s10994-011-5273-4.
41. Marisa, L.; de Reyniès, A.; Duval, A.; Selves, J.; Gaub, M.P.; Vescovo, L.; Etienne-Grimaldi, M.-C.; Schiappa, R.; Guenot, D.; Ayadi, M.; et al. Gene Expression Classification of Colon Cancer into Molecular Subtypes: Characterization, Validation, and Prognostic Value. *PLOS Med.* **2013**, *10*, e1001453, https://doi.org/10.1371/journal.pmed.1001453.
42. Klambauer, G.; Schwarzbauer, K.; Mayr, A.; Clevert, D.-A.; Mitterecker, A.; Bodenhofer, U.; Hochreiter, S. cn.MOPS: mixture of Poissons for discovering copy number variations in next-generation sequencing data with a low false discovery rate. *Nucleic Acids Res.* **2012**, *40*, e69–e69, https://doi.org/10.1093/nar/gks003.
43. Benítez, A.A.; Bourqui, R.; Thébault, P.; Mougin, F. GSAn: an alternative to enrichment analysis for annotating gene sets. *NAR Genom. Bioinform.* **2020**, *2*, lqaa017, https://doi.org/10.1093/nargab/lqaa017.

44. Imrichova, H.; Hulselmans, G.; Atak, Z.K.; Potier, D.; Aerts, S. i-cisTarget 2015 update: generalized cis-regulatory enrichment analysis in human, mouse and fly. *Nucleic Acids Res.* **2015**, *43*, W57–W64, https://doi.org/10.1093/nar/gkv395.
45. Csárdi, G.; Nepusz, T. The Igraph Software Package for Complex Network Research. InterJournal 2006, Complex Systems, 1695.
46. Scrucca, L.; Fop, M.; T., B.M.; Adrian, E.R. mclust 5: Clustering, Classification and Density Estimation Using Gaussian Finite Mixture Models. *R J.* **2016**, *8*, 289–317, https://doi.org/10.32614/rj-2016-021.
47. Blokzijl, F.; Janssen, R.; Van Boxtel, R.; Cuppen, E. MutationalPatterns: comprehensive genome-wide analysis of mutational processes. *Genome Med.* **2018**, *10*, 33, https://doi.org/10.1186/s13073-018-0539-0.
48. Therneau, T.M. A Package for Survival Analysis in R 2020.
49. Kassambara, A.; Kosinski, M.; Biecek, P. Survminer: Drawing Survival Curves Using "Ggplot2" 2019.
50. Mootha, V.K.; Lindgren, C.M.; Eriksson, K.-F.; Subramanian, A.; Sihag, S.; Lehar, J.; Puigserver, P.; Carlsson, E.; Ridderstråle, M.; Laurila, E.; et al. PGC-1α-responsive genes involved in oxidative phosphorylation are coordinately downregulated in human diabetes. *Nat. Genet.* **2003**, *34*, 267–273, https://doi.org/10.1038/ng1180.
